# Estimating optimally tailored active surveillance strategy under interval censoring

**DOI:** 10.1093/biomtc/ujaf067

**Published:** 2025-05-30

**Authors:** Muxuan Liang, Yingqi Zhao, Daniel W Lin, Matthew Cooperberg, Yingye Zheng

**Affiliations:** Department of Biostatistics, University of Florida, Gainesville, FL 32611, United States; Public Health Sciences Division, Fred Hutchinson Cancer Center, Seattle, WA 98109, United States; Department of Urology, University of Washington, Seattle, WA 98195, United States; Epidemiology & Biostatistics, University of California, San Francisco, CA 94143, United States; Public Health Sciences Division, Fred Hutchinson Cancer Center, Seattle, WA 98109, United States

**Keywords:** cancer surveillance, decision-making, generalization error, interval censoring, missing data

## Abstract

Active surveillance (AS) using repeated biopsies to monitor disease progression has been a popular alternative to immediate surgical intervention in cancer care. However, a biopsy procedure is invasive and sometimes leads to severe side effects of infection and bleeding. To reduce the burden of repeated surveillance biopsies, biomarker-assistant decision rules are sought to replace the fix-for-all regimen with tailored biopsy intensity for individual patients. Constructing or evaluating such decision rules is challenging. The key AS outcome is often ascertained subject to interval censoring. Furthermore, patients will discontinue participation in the AS study once they receive a positive surveillance biopsy. Thus, patient dropout is affected by the outcomes of these biopsies. This work proposes a non-parametric kernel-based method to estimate a tailored AS strategy’s true positive rates (TPRs) and true negative rates (TNRs), accounting for interval censoring and immediate dropouts. We develop a weighted classification framework based on these estimates to estimate the optimally tailored AS strategy and further incorporate the cost-benefit ratio for cost-effectiveness in medical decision-making. Theoretically, we provide a uniform generalization error bound of the derived AS strategy, accommodating all possible trade-offs between TPRs and TNRs. Simulation and application to a prostate cancer surveillance study show the superiority of the proposed method.

## 1 INTRODUCTION

Active surveillance (AS) has become a widely used alternative to immediate aggressive interventions such as surgery for managing low-grade cancer (Ganz et al., 2012; Cooperberg and Carroll, 2015; Chen et al., 2016; Auffenberg et al., 2017; Sanda et al., 2018). It involves periodic tumor monitoring with invasive tests such as biopsies, often following a one-size-fits-all schedule for all patients. To reduce the burden of frequent testing, biomarker-assistant rules are sought to personalize AS intervals based on patients’ characteristics. However, creating these rules and evaluating their clinical validity remain challenging due to the dynamic nature of AS and how the key AS outcome is ascertained.

Our research is motivated by the Canary Prostate Active Surveillance Study (PASS), a multicenter, prospective cohort study enrolling men with low-grade prostate cancer opting for AS (Cooperberg et al., 2020). In PASS, patients are closely monitored for disease progression, with prostate-specific antigen (PSA) tests every 3 months, clinical visits every 6 months, and ultrasound-guided biopsies at 6, 12, and 24 months after diagnosis, then every 2 years. A key goal is to develop an optimally tailored AS dynamic regimen. The outcome of AS, disease progression, indicated by reclassification to clinically significant cancer, is determined through biopsies, with its timing known only between the last negative and the most recent positive biopsy. The patient typically drops out of the study after reclassification. Deriving and evaluating the AS rule need to account for the interval censoring and immediate dropouts.

Many model-based approaches have been proposed to estimate the covariate effects on interval-censored events. Parametric and semiparametric maximum likelihood estimators and sieve likelihood estimators address interval censoring under proportional hazards models (Huang, 1995; 1996; Rossini and Tsiatis, 1996; Huang and Rossini, 1997; Goggins and Finkelstein, 2000; Wang and Dunson, 2011; Zeng et al., 2017; Gao et al., 2019), as well as additive hazard and accelerated failure time models (Lin et al., 1998; Shiboski, 1998; Shen, 2000; Martinussen and Scheike, 2002; Tian and Cai, 2006; Lin and Wang, 2010). To construct surveillance rules with longitudinal measurements, joint modeling or partly conditional models are adapted with these baseline models to account for interval-censored outcomes (Tsiatis and Davidian, 2004; Yu et al., 2008; Maziarz et al., 2017; Tomer et al., 2019). However, these methods depend on specific assumptions, and their performance can be sensitive to them, while also requiring significant computational resources (eg, expectation-maximization algorithms) (Mongoué-Tchokoté and Kim, 2008; McMahan et al., 2013). Thus, a robust treatment for the interval-censored event under a more flexible and computationally efficient framework would broaden the applicability of the developed rules.

Chan et al. (2021) proposed non-parametric estimators for time-dependent true positive rate (TPR) and true negative rate (TNR) via kernel regressions to evaluate the prediction performance of a baseline risk score when the occurrence of a particular clinical condition is only examined at the scheduled visit. Their estimators are model-agnostic and computationally simple but assume random dropouts and panel current status data, which may not hold in surveillance studies where patients often leave after disease progression is detected. In addition, their focus was not on deriving a decision rule. Our shift from a linear risk score to a surveillance rule represents a more actionable and clinically interpretable framework for decision-making. To this end, we follow classification-based approaches in deriving decision rules for medical decision-making. Dong et al. (2023) introduced a framework incorporating time-dependent TPR and TNR into the objective function for learning optimal dynamic surveillance rules, accommodating right-censored outcomes through inverse-censoring-probability weighting (IPCW). However, this method does not directly address interval-censored outcomes, which are common in settings with infrequent diagnostic procedures.

In this work, we develop a flexible framework that can handle interval-censored events and non-random dropouts with computationally efficient algorithms for surveillance rule derivation. We make two major contributions. First, different from Chan et al. (2021), we propose a two-dimensional kernel function for non-parametric TPR and TNR estimators to handle interval censoring and non-random dropouts simultaneously. Second, based on the classification framework of Dong et al. (2023), we construct a kernel-based benefit value function using proposed non-parametric TPR and TNR estimators to derive optimal AS strategies under the complex data structure of AS studies. In addition, the proposed benefit value function can incorporate cost-benefit ratios and disease prevalence as weights to target cost-effective decisions. Our proposed work may significantly broaden the framework’s applicability and overcome limitations present in the prior work.

## 2 Method

### 2.1 Weighted benefits value function and the optimality

Let $\boldsymbol {Z}_t$ represent the covariate information at time *t*, including baseline and time-invariant covariates, $\lbrace \boldsymbol {Z}_t\rbrace _{t\in \mathbb {R}_+}$ be a *p*-dimensional covariate process, and $\overline{\boldsymbol Z}_t$ represent the accrued covariate information up to *t*. Our goal is to derive a tailored AS decision rule, $d_s(\cdot )$, which maps $\overline{\boldsymbol Z}_s$, the accrued information up to the decision time point *s*, to a binary output $\lbrace 1,-1\rbrace$, with $d_s(\cdot ) = 1$ indicating a positive decision for conducting a future biopsy at $s+\tau$, and $d_s(\cdot ) = -1$ for a decision to skip the biopsy at that time. Here, $\tau$ is typically predetermined by the study protocol fixed for everyone. Therefore, $d_s(\cdot )$ leads to a surveillance intensity tailored to the individual’s covariate history. In particular, for ease of implementation and stable estimation given a typical limited study cohort size, we are interested in the stabilized strategy $d_0(\cdot )$, that is, $d_s(\overline{\boldsymbol Z}_s)=d_0(\boldsymbol {Z}_s)$. A stabilized strategy shares the same format at different time points *s*, and takes only the most up-to-date covariate information as input.

The validity of $d_s(\cdot )$, that is, whether a biopsy should be scheduled at time $s+\tau$, depends on whether a surveillance endpoint will occur within the time window $[s, s+\tau ]$. For any tailored AS rule, we first define a weighted benefits value function based on the TPR and the TNR (Dong et al., 2023). At a landmark time point *s*, pertinent to the outcome by a future time $s+\tau$, the time-varying TPR and TNR for a tailored AS strategy $d_s(\overline{\boldsymbol Z}_s)$ are defined as $\text{TPR}(d_s;s,\tau )=P\left\lbrace d_s(\overline{\boldsymbol Z}_s)=1\mid s < T\le s+\tau \right\rbrace $ and $\text{TNR}(d_s;s,\tau )=P\left\lbrace d_s(\overline{\boldsymbol Z}_s)=-1\mid T > s+\tau \right\rbrace ,$ where *T* is the event time, that is, the time of progression.

The $\text{TPR}(d_s;s,\tau )$ is the proportion of positive decisions among patients with an AS event occurs within time interval $(s,s+\tau ]$; the $\text{TNR}(d_s;s,\tau )$ is the proportion of negative decisions among patients who are event-free by $s+\tau$. Both high $\text{TPR}(d_s;s,\tau )$ and $\text{TNR}(d_s;s,\tau )$ are desirable for meaningful clinical decisions, but there is often a tradeoff between the two. We therefore define the time-specific weighted benefits value function at time point *s* as $\phi (d_s;s,\xi (s),\tau )=\text{TPR}(d_s;s,\tau ) +\xi (s) \text{TNR}(d_s;s,\tau ),$ where $\xi (s)$ is a pre-specified scalar representing the trade-off between $\text{TPR}(d_s;s,\tau )$ and $\text{TNR}(d_s;s,\tau )$. To obtain a dynamic regimen over time, we define the weighted benefits value function by averaging time-specific value functions over all landmark time points. Let $S(t)$ be the distribution function of the time making biopsy decisions. The value function is then defined as $\Phi \left(\boldsymbol d;\xi ,\tau \right):=\int \phi \lbrace d_t;t,\xi (t),\tau \rbrace \mathrm{d} S(t),$ where $\boldsymbol d=\left\lbrace d_s\right\rbrace _{s\ge 0}$.

Based on the definition of the weighted benefits value function, the optimally tailored AS regimen under a specific $\xi (\cdot )$ is defined as its maximizer, that is, $\boldsymbol d_{\xi ,\tau }:=\arg \max \Phi (\boldsymbol d;\xi ,\tau ).$ When the biopsy decisions have to be made at fixed landmark decision time points denoted as $0\le t_1 < t_2 < \cdots < t_J$, the value function $\Phi (\boldsymbol d;\xi ,\tau )=J^{-1}\sum _{j=1}^J \phi \lbrace d_{t_j};t_j,\xi (t_j),\tau \rbrace .$ If we are interested in the stabilized decision rule, the weighted benefits value function can be written as $\Phi (d_0;\xi ,\tau )=J^{-1}\sum _{j=1}^J \phi \lbrace d_0;t_j,\xi (t_j),\tau \rbrace .$

There are many possible choices of $\xi (\cdot )$. One possible choice is a $\xi (s)$ that characterizes the cost-benefit trade-offs. In this case, a strategy is cost-effective at time *s* if the number of unnecessary biopsies a patient can afford to catch an event (disease progression) is lower than an expected number, referred to as *r* (Pepe et al., 2016). It can be achieved by choosing $\xi (s)=\lbrace 1-\rho (s;\tau )\rbrace /\lbrace \rho (s;\tau ) r\rbrace$, where $\rho (s;\tau )=P\left(s < T\le s+\tau \mid T>s\right)$. Under this choice, given a fixed *r* and a strategy $\boldsymbol d$, the value function is equivalent to the difference between the number of unnecessary biopsies patients can afford and the number of unnecessary biopsies under the strategy *d* to catch an event. Thus, given a fixed *r*, a higher value function indicates better cost-benefit trade-offs, that is, fewer unnecessary biopsies compared with the number of unnecessary biopsies patients can afford to catch an event.

### 2.2 Estimating optimally tailored regimen under interval censoring and immediate dropouts

In this section, we consider estimating the time-varying TPR/TNR and the optimally tailored AS strategy using the observed data. First, we introduce our notations and assumptions.

Denote the event and censoring times as *T* and *C*, respectively. In the observed data, we do not directly observe *T*; instead, physicians would schedule *K* biopsies at times $\boldsymbol {N}=(N_1,\cdots , N_K)$, where $N_1 < \cdots < N_K$, to check whether disease progression occurs. Given these biopsy time points, without missing data or dropouts, we observe $\boldsymbol {\Delta }=(\Delta _1,\cdots , \Delta _K)$, where $\Delta _k=1\lbrace T\le N_k\rbrace$ indicating whether the disease progressed before the *k*th biopsy. However, we may be unable to observe $\Delta _k$ and $N_k$ due to lost-to-follow-up before the event time (censoring), missed biopsy appointments, and dropout due to disease progression. Specifically, to account for possible missed biopsy appointments, we use $\boldsymbol {\delta }=(\delta _1, \cdots , \delta _K)$ to indicate the completeness of the biopsy sequence, where $\delta _k=1$ indicating information on the *k*th biopsy, as well as $\Delta _k$, is available. To account for the censoring before the event time, let $\boldsymbol {\zeta }=(\zeta _1\cdots , \zeta _K)$, where $\zeta _k=1\lbrace C>N_k\rbrace$ indicating whether the censoring time is later than the *k*th biopsy time, that is, the *k*th biopsy is not censored; if $\zeta _k=0$, we cannot observe the *k*th biopsy, $N_k$ nor $\Delta _k$. In addition, we assume that the patient will drop out of the study immediately after $\Delta _k=1$. Under these notations, in our observed data, we can observe $N_k$ and $\Delta _k$ if and only if $\zeta _k\delta _k=1$ and $\Delta _{k^{\prime }}\delta _{k^{\prime }}=0$ for all $k^{\prime }< k$. We assume that $\overline{\boldsymbol Z}_s$ is available up to the time of the last biopsy.

For $\boldsymbol {N}$, $\boldsymbol {\zeta }$, and $\boldsymbol {\delta }$, we adopt the same assumptions as those in Chan et al. (2021). We assume that $\boldsymbol {N}$ is a random vector as patients may visit at random times near the scheduled visits, that is, the biopsy times $\boldsymbol {N}$ are independent of both *T* and $\lbrace \boldsymbol {Z}_t\rbrace _{t\in \mathbb {R}_+}$; the $P(\delta _k=1\mid \boldsymbol {\Delta }, \boldsymbol {N}, \lbrace \boldsymbol {Z}_t\rbrace _{t\in \mathbb {R}_+})=\rho _k>0$; the censoring indicator $P(\zeta _k=1\mid \boldsymbol {\Delta }, \boldsymbol {N}, \lbrace \boldsymbol {Z}_t\rbrace _{t\in \mathbb {R}_+})=\widetilde{\rho }_k>0$. The key difference between the settings in Chan et al. (2021) and ours is whether the patient will drop out from the study immediately after $\Delta _k=1$. For settings in Chan et al. (2021), the patients may still return to the study after $\Delta _k=1$; for surveillance study, the patients often drop out from the study and seek other medical interventions once $\Delta _k=1$ for some *k*.

Next, we propose an estimation method of the time-varying $\text{TNR}(d_s;s,\tau )$ based on the observed data under a tailored AS strategy, $d_s$. Following the approach in Chan et al. (2021), we can construct a non-parametric estimation for time-varying $\text{TNR}(d_s;s,\tau )$ for a given decision rule $d_s$. The key idea is to leverage the randomness of the biopsy time. Given an interval $(s, s+\tau ]$, suppose that we want to infer $P(s< T\le s+\tau )$, since the biopsy time is random, there are chances that the biopsy times are close to *s* or $s+\tau$; and thus by results of biopsies near *s* and $s+\tau$, we can infer $P(s< T\le s+\tau )$. By combining the biopsy information across biopsy times close to *s* or $s+\tau$, we can estimate the TPR/TNR.

Define $F_a(t;s)=P\left\lbrace d_s(\overline{\boldsymbol Z}_s)=a, T>t\right\rbrace ,$ where $a=\lbrace 1,-1\rbrace$. The $\text{TNR}(d_s;s)$ can be re-formulated as a function of $F_a(t;s)$, that is, $\text{TNR}(d_s;s)=F_{-1}(s+\tau ;s)\lbrace F_{-1}(s+\tau ;s)+F_1(s+\tau ;s)\rbrace ^{-1}.$ Following Chan et al. (2021), we consider the following estimation for $\text{TNR}(d_s;s)$, that is, $\widehat{\text{TNR}}(d_s;s)=E_n[1\lbrace d_s(\overline{\boldsymbol Z}_s)=-1\rbrace W_{-1,s+\tau }],$ where $W_{-1,t}=\lbrace \sum _k(1-\Delta _k)\zeta _k\delta _kK_h(N_k-t)\rbrace \left[\sum _kE_n\lbrace (1-\Delta _k)\zeta _k\delta _kK_h(N_k-t)\rbrace \right]^{-1},$ the function $K_h(\cdot )=h^{-1}K(\cdot /h)$ and $K(\cdot )$ is a univariate kernel function, and *h* is the bandwidth. The $E_n(\cdot )$ denotes the sample average of the subjects whose last biopsy is after $s+\tau$. The proposed estimator utilizes all observed negative biopsies. Although we do not observe future positive biopsy results after a positive biopsy, we observe all negative biopsies except those that are missing or censored. Thus, the proposed estimator for $\text{TNR}(d_s;s,\tau )$ is also expected to be consistent in our setting.

However, estimating $\text{TPR}(d_s;s,\tau )$ is nontrivial. In our setting, patients immediately drop out from the study once $\Delta _k=1$ for some *k*, and thus, the positive biopsy times after the first positive biopsy cannot be observed. Directly using the estimator in Chan et al. (2021) for TPRs leads to a biased estimation since whether we can observe a positive biopsy also depends on previous biopsy results. To address the immediate dropouts, we consider adjacent negative–positive pairs of biopsies. We say an adjacent pair of biopsies is a negative–positive pair if and only if $\Delta _{(k)}=0$ and $\Delta _k=1$, where $(k)$ is the index of the adjacent observed biopsy before the *k*th biopsy. Different from the positive biopsies, whether an adjacent negative–positive pair will be observed does not depend on the past biopsy results; the adjacent negative-positive pair will always be observed if there is no censoring or missing, and thus is not affected by the immediate dropouts. Thus, these pairs can always inform the shortest interval identifiable from the observed data that contains the event time, that is, $T\in (N_{(k)}, N_k]$. In addition, since the biopsy times are random, the biopsy times of adjacent biopsy pairs are random. Given a time interval of interest, the frequency of adjacent negative–positive pairs with biopsy times similar to the time interval of interest can inform the prevalence of an event; thus, adjacent negative–positive pairs can help address the problem of interval censoring. Denote $N_0=0$ and $\zeta _0\delta _0=1$, corresponding to the confirmatory biopsy or baseline diagnosis. Theorem 1 shows that the $P\left\lbrace d_s(\overline{\boldsymbol Z}_s)=a, s \le T\le s+\tau \right\rbrace$ is identifiable using observed adjacent negative–positive pairs. Its proof is in the online Supplementary Material.

Theorem 1: For any *k* and *s*, we have

\begin{eqnarray*}
&& P\lbrace d_s(\overline{\boldsymbol Z}_s)=a, s \le T\le s+\tau \rbrace \\
&&\quad =P\lbrace d_s(\overline{\boldsymbol Z}_s)=a, \Delta _{(k)}=0, \Delta _k=1\mid N_{(k)}=s,\\
&&\qquad N_k=s+\tau , \delta _k\zeta _k=1\rbrace ,
\end{eqnarray*}

where $(k)$ is the index of the adjacent observed biopsy before the *k*th biopsy.

Following Theorem 1, for any *k*, notice that


\begin{eqnarray*}
\text{TPR}(d_s;s,\tau )&=&P\lbrace d_s(\overline{\boldsymbol Z}_s)=1, \Delta _{(k)}=0,\\
&&\quad \Delta _k=1, \delta _k\zeta _k=1\mid N_{(k)}=s, N_k=s+\tau \rbrace \\
&&\times \,P^{-1}\lbrace \Delta _{(k)}=0, \Delta _k=1,\\
&&\quad \delta _k\zeta _k=1\mid N_{(k)}=s,
N_k=s+\tau \rbrace .
\end{eqnarray*}

Thus, the $\text{TPR}(d_s;s,\tau )$ can be then estimated by $\widehat{\text{TPR}}(d_s;s,\tau ) =E_n[1\lbrace d_s(\overline{\boldsymbol Z}_s)=1\rbrace W_{1,s}],$ where $W_{1,s}=\lbrace \sum _k \Delta _k(1-\Delta _{(k)})\zeta _k\delta _k\widetilde{K}_{\widetilde{h}}(N_k-s-\tau , N_{(k)}-s)\rbrace [\sum _k E_n\lbrace \Delta _k(1-\Delta _{(k)})\zeta _k\delta _k\widetilde{K}_{\widetilde{h}}(N_k-s-\tau , N_{(k)}-s)\rbrace ]^{-1},$ the function $\widetilde{K}_{\widetilde{h}}(t_1,t_2)=\widetilde{h}^{-2}\widetilde{K}(t_1/\widetilde{h},t_2/\widetilde{h})$, $\widetilde{K}(\cdot ,\cdot )$ is a two-dimensional kernel function, and $\widetilde{h}$ is the associated bandwidth that could be different from *h*.

Based on the estimators of $\text{TNR}(d_s;s,\tau )$ and $\text{TPR}(d_s;s,\tau )$, we can estimate the optimally tailored AS strategy. For the simplicity of the notation, we only consider the strategy with a stabilized decision rule in the following discussion. For stabilized decision rules, we can maximize $\widehat{\Phi }_n(d_0;\xi ,\tau )=J^{-1}\sum _j E_n[1\lbrace d_{0}(\boldsymbol {Z}_{t_j})=1\rbrace W_{1,t_j}+1\lbrace d_{0}(\boldsymbol {Z}_{t_j})=-1\rbrace \xi (t_j)W_{-1,t_j+\tau }].$

Remark 1: The proposed method assumes that $\boldsymbol N$ is independent of *T* and $\lbrace \boldsymbol Z_t\rbrace _{t\in R_{+}}$. When such an assumption does not hold, estimation will be biased. Under a less stringent assumption that $T\mid \overline{\boldsymbol Z}_s, N_k=s, N_{k^{\prime }}=s+\tau$ has the same distribution as $T\mid \overline{\boldsymbol Z}_s$ for every *s, k* and $k^{\prime }$, our proposed method for estimating TPR is still valid if we modify the kernel weights by including $\overline{\boldsymbol Z}_s$,

\begin{eqnarray*}
W_{1,s}(\overline{\boldsymbol z}_s)&=&\left[\sum _k E_n\lbrace \Delta _k(1-\Delta _{(k)})\zeta _k\delta _k\widetilde{K}_{\widetilde{h}}(N_k-s-\tau , N_{(k)}-s, \overline{\boldsymbol Z}_s-\overline{\boldsymbol z}_s)\rbrace \right] \\
&&\times \,\left[\sum _k E_n\lbrace \zeta _k\delta _k\widetilde{K}_{\widetilde{h}}(N_k-s-\tau , N_{(k)}-s, \overline{\boldsymbol Z}_s-\overline{\boldsymbol z}_s)\rbrace \right]^{-1},
\end{eqnarray*}

and $\widehat{\text{TPR}}(d_s;s,\tau ) =E_n[1\lbrace d_s(\overline{\boldsymbol Z}_s)=1\rbrace W_{1,s}(\overline{\boldsymbol Z}_s)]/E_n\lbrace W_{1,s}(\overline{\boldsymbol Z}_s)\rbrace$. In addition, when $\boldsymbol N$ follows a discrete distribution over landmark decision time points, we can choose discrete kernel functions instead of a continuous kernel function (Rajagopalan and Lall, 1995).

### 2.3 Computationally efficient algorithms

Maximizing the weighted benefits value function is equivalent to solving a weighted classification problem, that is, $\min _{\boldsymbol d} J^{-1}\sum _j E_n[1\lbrace d_{0}(\boldsymbol {Z}_{t_j})\not=1\rbrace W_{1,t_j}+1\lbrace d_{0}(\boldsymbol {Z}_{t_j})\not=-1\rbrace \xi (t_j)W_{-1,t_j+\tau }].$

To prevent the complication of optimizing an objective function that includes the indicator function, we substitute it with a convex surrogate loss function, denoted as $\phi$, and consider


(1) \begin{eqnarray*}
\min _{f\in \mathcal {F}} \ell _{\phi ,n}(f;\xi , \lambda _n)
&=&J^{-1}\sum _j E_n[W_{+,t_j}\phi \lbrace f(\boldsymbol {Z}_{t_j})\rbrace \\
&&+\,W_{-,t_j}\phi \lbrace -f(\boldsymbol {Z}_{t_j})\rbrace]+\lambda _n\Vert f\Vert _{\mathcal {F}}^2, \\
\end{eqnarray*}

where $W_{+,t_j}=\left\lbrace W_{1,t_j}-\xi (t_j)W_{-1,t_j+\tau }\right\rbrace _{+}$ and $W_{-,t_j}=\left\lbrace W_{1,t_j}-\xi (t_j)W_{-1,t_j+\tau }\right\rbrace _{-}$, $\mathcal {F}$ is a pre-specified function class in a Hilbert space, and $\Vert \cdot \Vert _{_{\mathcal {F}}}$ is the associated norm. The penalization $\lambda _n\Vert f\Vert _{\mathcal {F}}^2$ is added to avoid over-fitting, where $\lambda _n$ is a tuning parameter. Denote its minimizer as $\widehat{f}_{\xi ,\lambda _n}$; the estimated AS strategy can be characterized by $\widehat{d}_{\xi ,\lambda _n}(\boldsymbol {Z}_t)=\mathrm{sgn}\left\lbrace \widehat{f}_{\xi ,\lambda _n}(\boldsymbol {Z}_t)\right\rbrace$.

To account for cost-benefit ratios, we set $\xi (s)=\lbrace 1-\rho (s;\tau )\rbrace /\lbrace \rho (s;\tau ) r\rbrace$. For constructing the objective function, it’s necessary to estimate $\xi (s)$. In the online Supplementary Material, we derive an estimator using techniques similar to those for constructing $\widehat{\text{TPR}}(d_0;s,\tau )$. Denote the estimated $\xi (s)$ as $\widehat{\xi }(s)$, and we then minimize $\ell _{\phi ,n}(f;\widehat{\xi }, \lambda _n)$ over $f\in \mathcal {F}$. Denote its minimizer as $\widehat{f}_{\widehat{\xi }, \lambda _n}$; the estimated AS strategy is defined by $\widehat{d}_{\widehat{\xi },\lambda _n}(\boldsymbol {Z}_t)=\mathrm{sgn}\left\lbrace \widehat{f}_{\widehat{\xi },\lambda _n}(\boldsymbol {Z}_t)\right\rbrace$.

Minimizing $\ell _{\phi ,n}(f;\xi , \lambda _n)$ fundamentally resolves a weighted classification problem via penalized empirical risk minimization. As $\ell _{\phi ,n}(f;\xi , \lambda _n)$ is convex in *f*, we can employ the gradient-based approaches for its solution. In our implementation, $\phi$ is chosen as the logistic loss with linear decision rules (i.e., $f(\cdot )$ has a linear form), and thus minimizing $\ell _{\phi ,n}(f;\xi , \lambda _n)$ is the same as a weighted logistic regression with a ridge penalty. Existing R packages, for example, *glmnet*, can be used to implement the proposed method. We refer to our proposed method as the Optimization with the Surrogate Function approach for Interval-censored data (OSF-I).

## 3 Theoritical properties

In this section, we state the theoretical properties of the proposed estimators under a stabilized decision rule. The detailed proof of the main theorem can be found in the online Supplementary Material. The theoretical properties of the time-varying surveillance decision rules are implied in the proof. To start with, given a decision rule $d_0$, we define ${\Phi }(d_0;\xi ,\tau )=J^{-1}\sum _j \left\lbrace \text{TPR}(d_0;t_j,\tau )+\xi (t_j)\text{TNR}(d_0;t_j,\tau )\right\rbrace .$ To assess the theoretical property of the tailored AS rule under $\widehat{d}_{\xi , \lambda _n}$, we use a generalization error that compares ${\Phi }(\widehat{d}_{\xi , \lambda _n};\xi ,\tau )$ with the optimally tailored AS dynamic regimen. The optimally tailored AS dynamic regimen at time $t_j$ is defined as the maximizer of $\text{TPR}(d_{t_j};t_j,\tau )+\xi (t_j)\text{TNR}(d_{t_j};t_j,\tau )$. Denote the maximizer at time $t_j$ as $d^{*}_{\xi , j}$, and define $\Phi ^{*}(\xi ,\tau )=J^{-1}\sum _j \left\lbrace \text{TPR}(d^{*}_{\xi , j};t_j,\tau )+\xi (t_j)\text{TNR}(d^{*}_{\xi , j};t_j,\tau )\right\rbrace .$ The generalization error is then defined as $\Phi (\widehat{d}_{\xi , \lambda _n};\xi ,\tau )-{\Phi }^{*}(\xi ,\tau ).$ To accommodate the case where $\xi$ is chosen using the cost-benefit ratio, we derive an upper bound for the generalization error $\left\lbrace \Phi (\widehat{d}_{\xi , \lambda _n};\xi ,\tau )-{\Phi }^{*}(\xi ,\tau )\right\rbrace$ which is uniformly held for $\xi \in \Xi :=[\underline{\xi },\overline{\xi }]^J$, where $\underline{\xi }$ is some constant bounded away from 0 and $\overline{\xi }$ is some constant bounded away from $+\infty$.

For the function class $\mathcal {F}$, we impose a complexity constraint regarding the covering number of the space $\mathcal {F}$. The covering number $N\lbrace \epsilon , \mathcal {F}, L_2(P)\rbrace$ is defined as the minimal number of closed $L_2(P)$-balls of radius $\epsilon >0$ required to cover $\mathcal {F}$, where $\Vert f\Vert _{P,2}^2=E(f^2)$ (Van de Geer, 2008). Under these notations, we assume the following:

Assumption 1: There exist constants $0 < v < 2$ and *c* such that $\forall \epsilon \in (0,1]$, we have $\sup _P\log N\left\lbrace \epsilon , \mathcal {F}, L_2(P)\right\rbrace \le c\epsilon ^{-v}$, where the supremum is taken over all finitely discrete probability measures *P*.

Assumption 2: The kernel function $K(\cdot )$ is a $\nu$th order univariate kernel function with a bounded 2nd order derivative and compact support; the kernel function $\widetilde{K}(\cdot , \cdot )$ is a $\nu$th order bivariate kernel function with a bounded 2nd order derivative and compact support.

Assumption 1 controls the complexity of the function class $\mathcal {F}$ and can be satisfied for many choices of function classes. For example, if $\mathcal {F}$ is a class of all linear combinations of elements in a fixed base class with a finite Vapnik–Chervonenkis dimension, Assumption 1 is satisfied according to Theorem 9.4 in Kosorok (2008). Assumption 2 contains commonly adopted assumptions for kernel regressions (Nadaraya, 1964). Assumptions on the surrogate loss $\phi$ can be found in the online Supplementary Material. Under these assumptions, our main theorem below provides a uniformly valid upper bound for the generalization error.

Theorem 2: Suppose that Assumptions 1 and 2 hold with $\lambda _n\rightarrow 0$, with probability approaching to 1, we have that $\Phi (\widehat{d}_{\xi , \lambda _n};\xi )-\Phi ^{*}(\xi )\lesssim J^{-1/s}C\left\lbrace \mathcal {A}(\lambda _n;\xi )+\lambda _n^{-1/2}\hbar \right\rbrace ^{1/s}$ uniformly holds for all $\xi \in \Xi$, where $\mathcal {A}(\lambda _n;\xi )$ is the approximation error due to the function class $\mathcal {F}$ (see formula in the online Supplementary Material), $\hbar =h^{\nu }+(nh)^{-1/2}+\widetilde{h}^{\nu }+(n\widetilde{h}^2)^{-1/2},$ and *s* is a positive constant depending on the choice of $\phi$.

The result in Theorem 2 shows an upper bound for the weighted benefits value difference between the estimated tailored AS rule and the optimally tailored AS dynamic regimen. To achieve the lowest generalization error, we can set *h* and $\widetilde{h}$ to minimize $\lambda _n^{-1/2}\hbar$; when $h=n^{-1/(2\nu +1)}$ and $\widetilde{h}=n^{-1/(2\nu +2)}$, the term $\lambda _n^{-1/2}\hbar$ is minimized for any given $\lambda _n$. In our simulation and real data, we specify $h=C_b n^{-1/5}$ and $\widetilde{h}=C_b n^{-1/6}$, where $C_b$ is some positive constant. To select the optimal $\lambda _n$ and $C_b$, we use the cross-validation procedure. From the uniform generalization error, if we adopt $\widehat{\xi }(\cdot )$ as $\xi (\cdot )$ in optimization (1), then we can provide a generalization error for $\Phi (\widehat{d}_{\widehat{\xi },\lambda _n};\xi ^{*})$ (see online Supplementary Material), which is the generalization error of the rule incorporating the cost-benefit ratio.

## 4 SIMULATIONS

In this section, we compare the proposed method for estimating TPR, TNR, and the tailored AS rule with other methods through simulations.

### 4.1 Data generation

The data-generating process is as follows. We first generate the underlying covariate with measurement error, that is,$X_l(t)=W_l(t)+\epsilon _l(t)$, where $W_l(t)=a_{0,l}+a_{1,l}\log (t/\nu )$ and $\boldsymbol {a}_{l,\cdot }=(a_{0,l},a_{1,l})$ are generated from a bivariate normal distribution with mean $(-0.1,-0.1)^\top$ and covariance matrix $(0.82^2, -0.005;-0.005, 0.13^2)$. The measurement errors $\epsilon _l(t)$ are independently generated from a mean-zero Gaussian distribution with a variance of 0.1. We generate the true event time, censoring time, and biopsy information following two scenarios. The censoring time *C* is generated from a uniform distribution on [12,150].

1. The true event time *T* follows a proportional hazard model $\lambda (t)=\lambda _0(t)\exp \lbrace -0.7W_2(t)+0.8W_3(t)-1.3W_4(u)\rbrace$, where the baseline hazard $\lambda _0(t)=t/\nu (t/\nu _{\rm scale})^{\nu _{\rm shape}-1}$ and $\nu =30$, $\nu _{\rm scale}=15$, and $\nu _{\rm shape}=1.4$.
2. The true event time *T* is generated from $12+\nu \left[\widetilde{T}\nu _{\rm shape}^{-1} \gamma \exp \lbrace -\boldsymbol {a}_{0,\cdot }^\top \boldsymbol {\beta }-r(a_{0,1}+a_{0,2})^2\rbrace \right]^{1/\gamma },$ where $\widetilde{T}$ follows a standard exponential distribution, $\gamma =\nu _{\rm shape}+\boldsymbol {a}_{1,\cdot }^\top \boldsymbol {\beta }+r(a_{1,1}+a_{1,2})^2$, $r=0.1$, $\nu =30$, $\nu _{\rm scale}=15$, and $\nu _{\rm shape}=1.4$.

Scenarios (1) and (2) differ in the distributions of *T*: Scenario (1) uses a linear log-hazard model in $W_l(t)$; Scenario (2) uses a non-linear model. Comparing the results in linear and non-linear settings allows us to test the robustness of the proposed method to non-linear terms. For both scenarios, biopsy time depends on the biopsy gap $T_{\text{gap}}$, which controls the frequency/intensity of the biopsies. The first biopsy is generated from a uniform distribution on $[12, 3T_{\text{gap}}]$. After the first biopsy time $N_1$, we generate the rest of the biopsies sequentially. The following biopsy $N_t$ is generated from a uniform distribution on $[N_{t-1}+T_{\text{gap}}, N_{t-1}+3T_{\text{gap}}]$ until $N_{t-1}+T_{\text{gap}}>150$, where $N_{t-1}$ is the previous biopsy time. Through this generation process, the first follow-up biopsy $N_1$ is ensured after 12 months of confirmatory biopsy $N_0=0$; the adjacent biopsies have a minimum gap of $T_{\text{gap}}$. Then, we generate $\boldsymbol {\Delta }=(\Delta _1,\cdots , \Delta _K)$, where $\Delta _k=1\lbrace T\le N_k\rbrace$; and $\boldsymbol {\zeta }=(\zeta _1\cdots , \zeta _k)$, where $\zeta _k=1\lbrace C>N_k\rbrace$.

### 4.2 Comparison between estimators of TPRs and TNRs

This section compares the proposed method for estimating TPRs and TNRs, referred to as “KR-I”, with two alternatives. We consider the IPCW method in Dong et al. (2023) (referred to as “IPCW”) and the method in Chan et al. (2021) (referred to as “KR-CS”). For the IPCW method, we use the Kaplan–Meier estimator for the censoring distribution and apply IPCW to estimate TPRs and TNRs.

**FIGURE 1:**
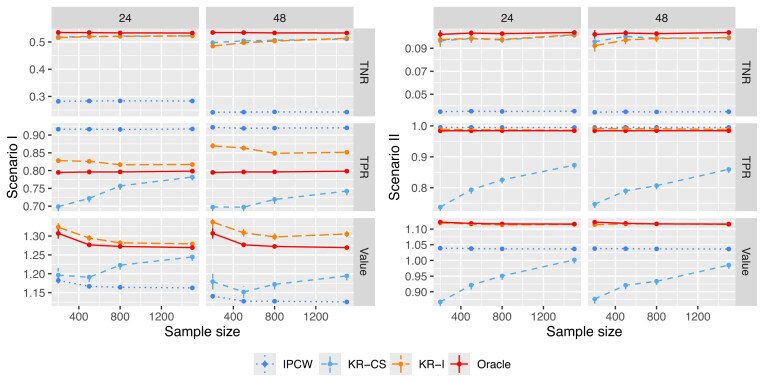
Estimating TNR, TPR, and weighted benefits value of a derived tailored AS rule using IPCW method in Dong et al. (2023) (“IPCW”), the method proposed in Chan et al. (2021) (“KS-CS”), and our proposed method (“KS-I”). The lines labeled as “Oracle” are the TPRs, TNRs, and values calculated using the true event time without censoring.

To assess the performance of different approaches, we estimate the TPRs and TNRs of a fixed surveillance rule using different methods. To derive the tailored AS rule and generate datasets to estimate TPRs and TNRs, we assume no missed biopsy and set the biopsy gap $T_{\text{gap}}=24$. We generate a dataset with a sample size of 500, and use the optimization with the surrogate function approach for right-censored data (referred to as “OSF-R”) in Dong et al. (2023) to derive a tailored AS rule (fixing $r=3$ in Scenario [1]; and $r=2$ in Scenario [2]). To evaluate this rule, we generate an independent dataset with sample sizes varying from 200 to 500 and implement our proposed KR-I, IPCW, and KR-CS approaches. When generating this dataset, we vary the biopsy gap $T_{\text{gap}}$ from 24 to 48. We use the true event time *T* to calculate the true TPRs, TNRs, and weighted benefits values of the derived tailored AS rule. The entire procedure is repeated 500 times. Figure 1 summarizes the results for Scenarios (1) and (2). In both scenarios, the proposed method achieves the most accurate estimates w.r.t the true TPRs, TNRs, and weighted benefits values.

### 4.3 Comparison between methods to estimate the tailored AS rule

We compare the proposed method (referred to as “OSF-I”) and the OSF-R approach to estimate the optimally tailored AS rule. The OSF-R approach minimizes the relaxation of the empirical objective, which is similar to our objective. However, the OSF-R treats the event time as the biopsy time subject to right-censoring and employs an IPCW method to account for it. As shown in Section 4.2, the IPCW method may lead to biased estimations in TPRs and TNRs, and thus, a biased estimation in the optimally tailored AS rule.

**FIGURE 2:**
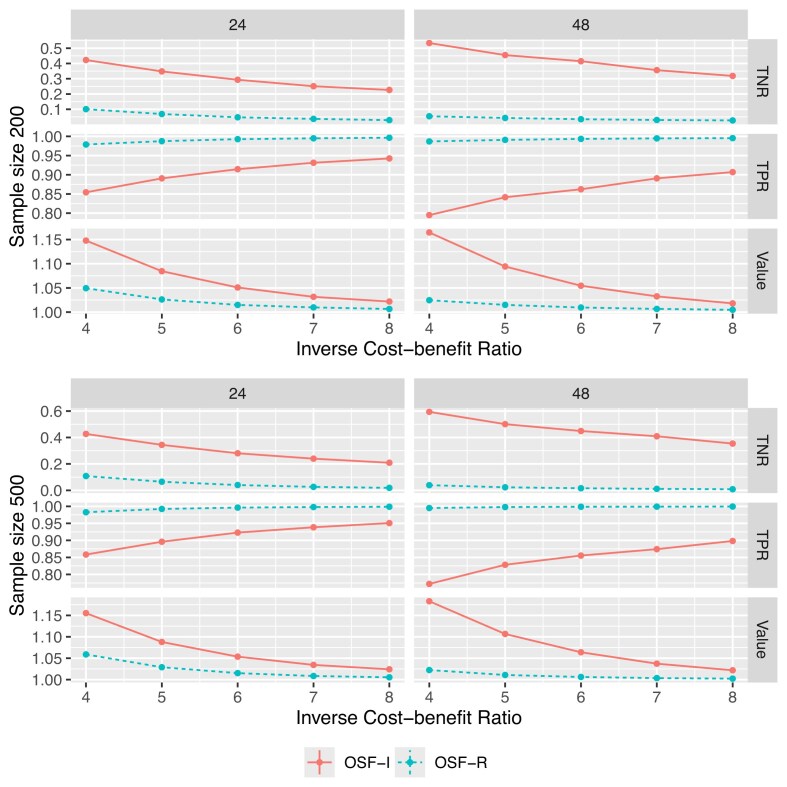
TNR, TPR, and weighted benefits value achieved by different methods under Scenario (1). The *x*-axis represents the inverse cost–benefit ratio, *r*, that is, how many unnecessary biopsies the patient can afford to catch an event (disease progression). The left and right columns summarize the results where $T_{\text{gap}}=24$ and $T_{\text{gap}}=48$, respectively.

**FIGURE 3:**
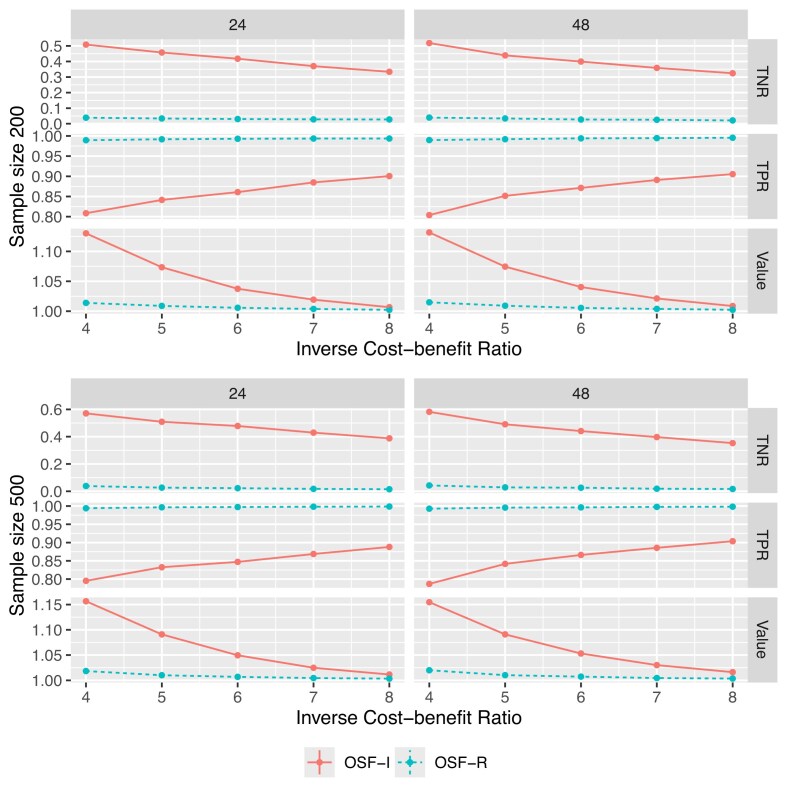
TNR, TPR, and weighted benefits value achieved by different methods under Scenario (2). The *x*-axis represents the inverse cost–benefit ratio, *r*, that is, the acceptable number of unnecessary biopsies to perform to catch an event (disease progression). The left and right columns summarize the results where $T_{\text{gap}}=24$ and $T_{\text{gap}}=48$, respectively.

For each scenario, we vary the sample size from 200 to 500, and the biopsy gap $T_{\text{gap}}$ from 24 to 48; we also vary the *r* from 2 to 8. The varies in the sample size, count of biopsy, $\xi$, and scenarios lead to a total of 32 simulation settings. We generate the training data for each simulation setting and estimate the decision rule on the training data; we repeat this procedure 500 times. We generate an independent testing dataset with a sample size of 1000 to compare different methods. On the testing dataset, we record the true event time *T*; thus, we can directly calculate the true TPR, TNR, and the value of the weighted net benefit for each derived AS rule. Figures 2 and 3 summarize the results for Scenarios (1) and (2), respectively. In both scenarios, compared to the OSF-R approach, the proposed OSF-I method achieves higher values for almost all choices of *r*. Compared with the settings where $T_{\text{gap}}=24$, in the settings where $T_{\text{gap}}=48$, the advantage of the proposed method is larger; this implies that the bias induced by treating the interval-censored data as right-censored increases with the increase of $T_{\text{gap}}$.

## 5 APPLICATION

We apply the proposed method to develop and evaluate a clinical decision rule for the tailored management of prostate cancer patients using data from PASS. We included 844 patients diagnosed since 2003 and enrolled in PASS before 2017, with Gleason grade group $(GG)$ 1 on diagnostic biopsy and GG1 or no tumor on confirmatory biopsy. The disease progression was defined as a reclassification, any increase in GG to 2 or more, detected through a surveillance biopsy. The predictors included the most recent PSA values, the most recent BMI status, the most recent prostate size, the PSA value at diagnosis, the most recent maximum core ratio, time since the confirmatory biopsy, and the counts of negative biopsies $(0,1,\ge 2)$.

We aim to derive an AS rule using updated information to decide whether a patient should receive a biopsy within 1 year ($\tau$ = 1 year). The time points of the decisions were chosen at $s=1,2,3,4$ years after the confirmatory biopsy. In the PASS study, the compliance rate was found to be very high, with patients adhering to their scheduled clinical visits under AS, and a surveillance biopsy is typically completed within a reasonable time window following the study protocol (Cooperberg et al., 2018). Therefore, the independent assumption regarding biopsy schedule $\boldsymbol N$ and progression time *T* appears reasonable. To compare different methods, we conduct two analyses. We repeatedly split the PASS cohort into equal training and testing sets in the first analysis. Each method is trained on the training set, and TPR, TNR, and weighted benefits values are calculated on the testing set. We report the mean and standard deviation over 100 repeats. In the second analysis, we train each method on the entire PASS cohort and use a cohort from the University of California San Francisco (UCSF) for external validation. The second analysis aims to validate the estimated surveillance strategies on external data. Confidence intervals are constructed by bootstrapping the UCSF dataset for $B=1000$. Table 1 summarizes the patient characteristics of two cohorts. Compared with the PASS cohort, the UCSF cohort is younger, more diverse, and has a higher event rate (grade reclassification). Additional results on other metrics can be found in the online Supplementary Material.

**TABLE 1:** Patient characteristics of the PASS and UCSF cohort.

Variable	PASS (844 patients)	UCSF (533 patients)
Age, No. (%), year		
$<$60	290 (34)	222 (42)
60–70	474 (56)	271 (51)
$>$70	80 (10)	40 (7)
BMI, median (IQR)	27 (25–30)	27 (25–29)
Race/ethnicity, No. (%)		
White	769 (91)	422 (79)
Black	42 (5)	12 (2)
Other	33 (4)	99 (19)
Diagnostic percent positive cores, median (IQR),%	8.3 (8.3–16.7)	11 (7–19)
No. missing percent positive cores at diagnosis	16	7
Diagnostic PSA, median (IQR), ng/mL	4.7 (3.5–6.4)	5.4 (4.2–7.3)
No. PSA measurements, median (IQR)	12 (7–19)	7 (4–13)
Most recent prostate size at confirmatory bx, median (IQR), mL	42 (30–58)	39 (30–54)
Grade reclassification, No. (%)	182 (22)	154 (29)
Follow-up since confirmatory bx, censored patients, median (IQR), y	3.2 (1.7–5.0)	2.5 (1.3–4.3)

**FIGURE 4:**
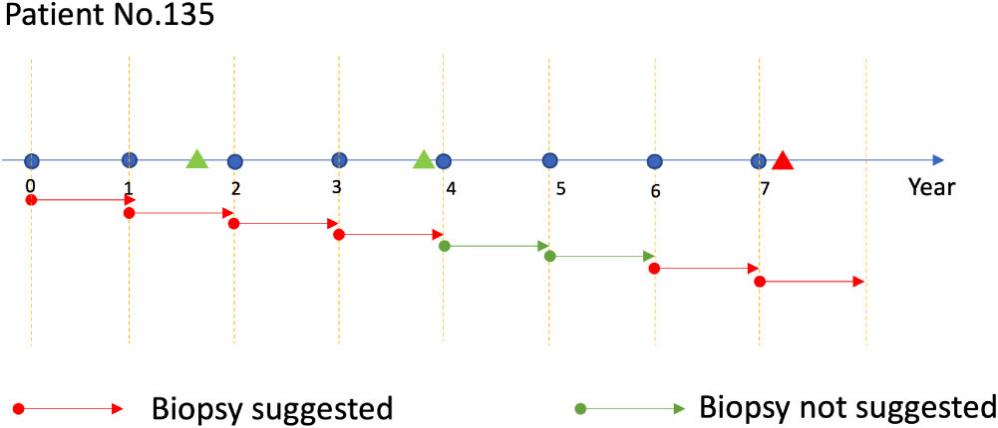
Visualization of the estimated AS strategy for Patient No.135. For this patient, 3 biopsies are recorded in the PASS data (the first two triangulars represent the biopsies that detect no disease progression; the final triangular represents the biopsy detecting disease progression), and there is a disease progression detected at Year 7.1. The circle points represent the time updating covariate information; the arrows below the time axis represent our estimated AS decisions at Years 0–7 (apply the stabilized AS decision rule derived from Years 1–4 in Years 0–7). Our estimated strategy suggests that the patient should skip biopsies at Years 4 and 5 due to the increased prostate size and resume the biopsies at Years 6 and 7 due to abnormally increased PSAs.

In both analyses, we set up a sequence of cost-benefit ratios ranging from 4 to 12. We consider a wide range of cost–benefit ratios reflecting varied emphasis on the TPR (increase from about 80% to higher than 98% when using the PASS cohort). We used the repeated sample-splitting strategy to compare different methods. Table 2 reports the results for both analyses. Using PASS only, the proposed OSF-I method achieves significantly higher values than the OSF-R method. When we use the UCSF data to validate the tailored AS rules derived by different methods, although the confidence intervals are wide, we can still observe that the proposed OSF-I method achieves higher values than the OSF-R method for most values of *r*. We further visualize our estimated rule to make biopsy decisions (see Figure 4).

**TABLE 2:** Comparisons using the PASS and UCSF data.

PASS only						
*r*		4	6	8	10	12
OSF-I	TPR	0.817 (0.0.802,0.833)	0.932 (0.922,0.941)	0.954 (0.945,0.962)	0.967 (0.959,0.974)	0.976 (0.969,0.983)
	TNR	0.399 (0.0.381,0.416)	0.201 (0.183,0.219)	0.137 (0.120,0.154)	0.100 (0.085,0.116)	0.078 (0.064,0.092)
	Value	1.318 (1.310,1.331)	1.100 (1.092,1.108)	1.040 (1.035, 1.045)	1.017 (1.014, 1.020)	1.009 (1.006, 1.011)
OSF-R	TPR	0.049 (0.037,0.061)	0.191 (0.170,0.211)	0.344 (0.318,0.369)	0.483 (0.460,0.506)	0.606 (0.584,0.627)
	TNR	0.984 (0.980,0.987)	0.934 (0.927,0.941)	0.861 (0.850,0.873)	0.772 (0.759,0.785)	0.676 (0.663,0.690)
	Value	1.162 (1.153,1.171)	0.896 (0.880,0.913)	0.832 (0.811,0.852)	0.833 (0.815,0.852)	0.862 (0.844,0.879)
PASS train + UCSF test						
*r*		4	6	8	10	12
OSF-I	TPR	0.788 (0.724,0.0.910)	0.847 (0.765,0.930)	0.940 (0.836,0.965)	0.955 (0.887,0.992)	0.955 (0.892,0.995)
	TNR	0.280 (0.213,0.0.334)	0.207 (0.136,0.254)	0.095 (0.084,0.185)	0.077 (0.050,0.126)	0.076 (0.042,0.119)
	Value	1.015 (0.908,1.126)	0.959 (0.850,1.028)	0.978 (0.879,1.007)	0.980 (0.914,1.016)	0.975 (0.911,1.011)
OSF-R	TPR	0.055 (0.015,0.112)	0.279 (0.193,0.376)	0.497 (0.348,0.552)	0.619 (0.510,0.718)	0.697 (0.595,0.791)
	TNR	0.971 (0.950,0.987)	0.816 (0.767,0.863)	0.619 (0.615,0.740)	0.502 (0.437,0.568)	0.412 (0.350,0.480)
	Value	0.728 (0.582,1.013)	0.660 (0.545,0.843)	0.718 (0.588,0.837)	0.762 (0.657,0.881)	0.795 (0.699,0.899)

## 6 DISCUSSION

This work proposes a weighted classification approach to estimate the optimal AS strategy. We utilize adjacent negative–positive pairs and employ two-dimensional kernel regressions for estimating TPRs and TNRs to accommodate the complications of interval-censored events and immediate dropouts. Existing methods for right-censored or panel status data have not fully addressed these complications.

Our work opens several avenues for future research. Longitudinal measures frequently involve missingness due to non-adherence, and methods like “last value carry-forward” for imputation may affect the optimality of AS strategies. Investigating ways to incorporate delayed or outdated information into strategy formulation is important. Our study relies on biopsies to identify disease progression. However, biopsies may have imperfect sensitivity or specificity and can be non-randomly ascertained in detecting progression. Addressing these challenges could enhance the robustness and applicability of AS strategies in clinical settings.

## Supplementary Material

ujaf067_Supplemental_Files Web Appendices, referenced in Sections 2 and 3, and R code implementing the proposed method are available with this paper at the Biometrics website on Oxford Academic.

## Data Availability

The data that support this paper’s findings are available by submitting the PASS Project Application at https://canarypass.org/researchers/. PASS specimens and data will be shared with investigators who have obtained appropriate approvals, for example, human subjects approval, data and material transfer agreement, and approval of the PASS Scientific Review Committee (SRC), and whose use is consistent with informed consent documents.

## References

[bib1] Auffenberg G. B., Lane B. R., Linsell S., Cher M. L., Miller D. C. (2017). Practice-vs physician-level variation in use of active surveillance for men with low-risk prostate cancer: implications for collaborative quality improvement. JAMA Surgery, 152, 978–980. 28636713 10.1001/jamasurg.2017.1586 PMC5831460

[bib2] Chan S., Wang X., Jazić I., Peskoe S., Zheng Y., Cai T. (2021). Developing and evaluating risk prediction models with panel current status data. Biometrics, 77, 599–609. 32562264 10.1111/biom.13317 PMC8168594

[bib3] Chen R. C., Rumble R. B., Loblaw D. A., Finelli A., Ehdaie B., Cooperberg M. R. et al. (2016). Active surveillance for the management of localized prostate cancer (Cancer Care Ontario Guideline): American Society of Clinical Oncology clinical practice guideline endorsement. Journal of Clinical Oncology, 34, 2182–2190. 26884580 10.1200/JCO.2015.65.7759

[bib4] Cooperberg M. R., Brooks J. D., Faino A. V., Newcomb L. F., Kearns J. T., Carroll P. R. et al. (2018). Refined analysis of prostate-specific antigen kinetics to predict prostate cancer active surveillance outcomes. European Urology, 74, 211–217. 29433975 10.1016/j.eururo.2018.01.017 PMC6263168

[bib5] Cooperberg M. R., Carroll P. R. (2015). Trends in management for patients with localized prostate cancer, 1990–2013. Journal of the American Medical Association, 314, 80–82. 26151271 10.1001/jama.2015.6036

[bib6] Cooperberg M. R., Zheng Y., Faino A. V., Newcomb L. F., Zhu K., Cowan J. E. et al. (2020). Tailoring intensity of active surveillance for low-risk prostate cancer based on individualized prediction of risk stability. JAMA Oncology, 6, e203187–e203187. 32852532 10.1001/jamaoncol.2020.3187 PMC7453344

[bib7] Dong X., Zheng Y., Lin D. W., Newcomb L., Zhao Y.-Q. (2023). Constructing time-invariant dynamic surveillance rules for optimal monitoring schedules. Biometrics, 79, 3895–3906. 37479875 10.1111/biom.13911 PMC10866138

[bib8] Ganz P. A., Barry J. M., Burke W., Col N. F., Corso P. S., Dodson E. et al. (2012). National Institutes of Health State-of-the-Science Conference: role of active surveillance in the management of men with localized prostate cancer. Annals of Internal Medicine, 156, 591–595. 22351514 10.7326/0003-4819-156-8-201204170-00401 PMC4774889

[bib9] Gao F., Zeng D., Couper D., Lin D. (2019). Semiparametric regression analysis of multiple right-and interval-censored events. Journal of the American Statistical Association, 114, 1232–1240. 31588157 10.1080/01621459.2018.1482756 PMC6777710

[bib10] Goggins W. B., Finkelstein D. M. (2000). A proportional hazards model for multivariate interval-censored failure time data. Biometrics, 56, 940–943. 10985240 10.1111/j.0006-341x.2000.00940.x

[bib11] Huang J. (1995). Maximum likelihood estimation for proportional odds regression model with current status data. Lecture Notes-Monograph Series, 27, 129–145.

[bib12] Huang J. (1996). Efficient estimation for the proportional hazards model with interval censoring. The Annals of Statistics, 24, 540–568.

[bib13] Huang J., Rossini A. (1997). Sieve estimation for the proportional-odds failure-time regression model with interval censoring. Journal of the American Statistical Association, 92, 960–967.

[bib14] Kosorok M. R. (2008). Introduction to Empirical Processes and Semiparametric Inference. New York: Springer.

[bib15] Lin D., Oakes D., Ying Z. (1998). Additive hazards regression with current status data. Biometrika, 85, 289–298.

[bib16] Lin X., Wang L. (2010). A semiparametric probit model for case 2 interval-censored failure time data. Statistics in Medicine, 29, 972–981. 20069532 10.1002/sim.3832

[bib17] Martinussen T., Scheike T. H. (2002). Efficient estimation in additive hazards regression with current status data. Biometrika, 89, 649–658.

[bib18] Maziarz M., Heagerty P., Cai T., Zheng Y. (2017). On longitudinal prediction with time-to-event outcome: comparison of modeling options. Biometrics, 73, 83–93. 27438160 10.1111/biom.12562 PMC5250577

[bib19] McMahan C. S., Wang L., Tebbs J. M. (2013). Regression analysis for current status data using the EM algorithm. Statistics in Medicine, 32, 4452–4466. 23761135 10.1002/sim.5863

[bib20] Mongoué-Tchokoté S., Kim J.-S. (2008). New statistical software for the proportional hazards model with current status data. Computational Statistics & Data Analysis, 52, 4272–4286.

[bib21] Nadaraya E. A. (1964). On estimating regression. Theory of Probability and Its Applications, 9, 141–142.

[bib22] Pepe M. S., Janes H., Li C. I., Bossuyt P. M., Feng Z., Hilden J. (2016). Early-phase studies of biomarkers: what target sensitivity and specificity values might confer clinical utility?. Clinical Chemistry, 62, 737–742. 27001493 10.1373/clinchem.2015.252163 PMC5003106

[bib23] Rajagopalan B., Lall U. (1995). A kernel estimator for discrete distributions. Journal of Nonparametric Statistics, 4, 409–426.

[bib24] Rossini A., Tsiatis A. (1996). A semiparametric proportional odds regression model for the analysis of current status data. Journal of the American Statistical Association, 91, 713–721.

[bib25] Sanda M. G., Cadeddu J. A., Kirkby E., Chen R. C., Crispino T., Fontanarosa J. et al. (2018). Clinically localized prostate cancer: AUA/ASTRO/SUO guideline. Part I: risk stratification, shared decision making, and care options. Journal of Urology, 199, 683–690. 29203269 10.1016/j.juro.2017.11.095

[bib26] Shen X. (2000). Linear regression with current status data. Journal of the American Statistical Association, 95, 842–852.

[bib27] Shiboski S. C. (1998). Generalized additive models for current status data. Lifetime Data Analysis, 4, 29–50. 9567054 10.1023/a:1009652024999

[bib28] Tian L., Cai T. (2006). On the accelerated failure time model for current status and interval censored data. Biometrika, 93, 329–342.

[bib29] Tomer A., Nieboer D., Roobol M. J., Steyerberg E. W., Rizopoulos D. (2019). Personalized schedules for surveillance of low-risk prostate cancer patients. Biometrics, 75, 153–162. 30039528 10.1111/biom.12940 PMC7380003

[bib30] Tsiatis A. A., Davidian M. (2004). Joint modeling of longitudinal and time-to-event data: an overview. Statistica Sinica, 14, 809–834.

[bib31] Van de Geer S. A. (2008). High-dimensional generalized linear models and the lasso. The Annals of Statistics, 36, 614–645.

[bib32] Wang L., Dunson D. B. (2011). Semiparametric Bayes’ proportional odds models for current status data with underreporting. Biometrics, 67, 1111–1118. 21175554 10.1111/j.1541-0420.2010.01532.x PMC3616323

[bib33] Yu M., Taylor J. M. G., Sandler H. M. (2008). Individual prediction in prostate cancer studies using a joint longitudinal survival–cure model. Journal of the American Statistical Association, 103, 178–187.

[bib34] Zeng D., Gao F., Lin D. (2017). Maximum likelihood estimation for semiparametric regression models with multivariate interval-censored data. Biometrika, 104, 505–525. 29391606 10.1093/biomet/asx029 PMC5787874

