# Supplementary material for: Estimating optimally tailored active surveillance strategy under interval censoring
Source: Biometrics. 2025 May 30;81(2):ujaf067. doi: 10.1093/biomtc/ujaf067 (PMC12123698; doi:10.1093/biomtc/ujaf067)
Supplement: ujaf067_Supplemental_Files — Web Appendices, referenced in Sections 2 and 3, and R code implementing the proposed method are available with this paper at the Biometrics website on Oxford Academic. [file ujaf067_supplemental_files.zip › Estimating_optimal_surveillance_rule_supplementmaterial.pdf]

BIOMETRICS 64, 1–14 DOI: 10.1111/j.1541-0420.2005.00454.x
December 2008

Supplementary Materials for "Estimating optimally tailored active surveillance strategy under interval censoring"

Muxuan Liang[1], Yingqi Zhao[2], Daniel W. Lin[3], Matthew Cooperberg[4], and Yingye Zheng[2,*]

[1]Department of Biostatistics, University of Florida, Gainesville, Florida, U.S.A.

[2]Public Health Sciences Division, Fred Hutchinson Cancer Center, Seattle, Washington, U.S.A.

[3]Department of Urology, University of Washington, Seattle, Washington, U.S.A.

[4]Epidemiology & Biostatistics, University of California, San Francisco, California, U.S.A.

**email:* yzheng@fredhutch.org

SUMMARY: In this online Supporting Information, we include the proofs of the theorems in the paper titled "Estimating optimal tailored active surveillance strategy under interval censoring."

KEY WORDS: Cancer Surveillance; Decision-making; Generalization error; Interval censoring; Missing data.

This paper has been submitted for consideration for publication in *Biometrics*

Web Appendix A

In this section, we discuss how to estimate the prevalence. In our formulation, we choose $\xi(s) = \{1 - \rho(s;\tau)\}/\{\rho(s;\tau)r\}$. For constructing the objective function, estimating $\xi(s)$ is necessary. We can derive an estimator for $\rho(s;\tau)$ by applying similar techniques used in constructing $\widehat{\text{TPR}}(d_0; s, \tau)$. Specifically,we construct $\widehat{\rho}(s;\tau)$ as follows:

$$\widehat{\rho}(s;\tau) = \frac{\sum_k E_n \left\{\Delta_k(1-\Delta_{(k)})\zeta_k\delta_k K_h(N_k - s - \tau, N_{(k)} - s)\right\}}{\sum_k E_n \left\{(1-\Delta_{(k)})\zeta_k\delta_k K_h(N_k - s - \tau, N_{(k)} - s)\right\}}.$$

Subsequently, we can derive an estimator $\widehat{\xi}(s) = \{1 - \widehat{\rho}(s;\tau)\}/\{\widehat{\rho}(s;\tau)r\}$, and minimize $\ell_{\phi,n}(f; \widehat{\xi}, \lambda_n)$ over $f \in \mathcal{F}$.

Web Appendix B

Both estimations of TPR and TNR involve kernel functions. Typical choices of kernel functions include the Gaussian kernel, Epanechnikov kernel, and other higher-order kernel functions (Silverman, 2018). In our implementation, we choose the Gaussian kernel function for its simplicity in computation. This section provides a thorough tuning strategy for kernel bandwidths in estimating TPRs, TNRs, and the optimal tailored active surveillance strategy. To reduce the number of parameters to be tuned, the one-dimensional kernel bandwidth is specified as $h = C\sigma n^{-1/5}$; the two-dimensional kernel bandwidth is specified as $\widetilde{h} = C\sigma n^{-1/6}$, where σ is the standard deviation of the biopsy time, n is the sample size at risk, i.e., number of the patients whose last biopsy is after time s. With this specification, our formula follows the rule of thumb provided in Silverman (2018); in addition, in our formula, we account for the decreasing number of patients due to decreasing compliance rates and possible time-varying variance of biopsy time.

To select the consistent C, we have separate procedures for estimating TPR and TNR vs. the optimal tailored active surveillance strategy. For the estimation of TPR and TNR, the key difficulty is that the true TPR and TNR are not directly observable. Thus, there is no

metric we can use to quantify the estimation errors of the estimates. To address this issue, we tune C based on the estimation of prevalence,i.e., $P(s < T \leqslant s+\tau \mid T > s)$. Based on the prevalence estimates, we design a loss function to select the appropriate C. Given a sequence of candidate C's, we first split the data (including only at-risk patients) into M folds. For each m, we then calculate the prevalence estimates $\widehat{P}(N_k < T < N_{k+1} \mid N_k, N_{k+1})$ using data excluding those in m-th fold. Then, on the m-th fold, we calculate the mean squared errors,

$$L_m(h) = \widehat{E}^{(m)}\left[\{\Delta_k(1-\Delta_{k+1}) - \widehat{P}(N_k < T < N_{k+1} \mid N_k, N_{k+1})\}^2\right].$$

Finally, we select the candidate C that leads to the smallest $M^{-1}\sum L_m(h)$. We then apply the selected C to estimate TPR and TNR. Notice that the optimal C is selected based on the at-risk set, i.e., the last biopsy time is after s; for different s, the optimal choice of C may be different to account for the decreased frequency of biopsies, especially when s is large.

To select the consistent C for the optimal tailored active surveillance strategy estimation, our procedure is more straightforward, given the reliable estimation approach for TPR and TNR. First, we split the entire data into m folds. Then we train our proposed method on the data, excluding m-th fold under each candidate choice of C. We calculate the value function using the estimated strategy using the data in m-th fold. The optimal choice of C is chosen to maximize the value function. In our implementation, for computation simplicity, we choose $M = 2$.

Web Appendix C

In this section, we provide an additional simulation setting where the frequency of biopsies decreases over time. The data generation of this simulation setting follows Scenario (2) in the main text. The major difference is that we generate the biopsies where the first two biopsies have a gap of $T_{\text{gap}} = 24$ and biopsies later have a time gap of $T_{\text{gap}} = 48$. Figure 1 summarizes

the performance of the proposed method and other comparison methods. Regarding estimating TNR, TPR, and weighted benefits value, our proposed method (with $r = 6$) performs better than other methods. In estimating the tailored AS rule, we can see that the proposed method achieves the highest value compared with other methods, similar to the results in Scenarios (1) and (2).

[Figure 1 about here.]

Web Appendix D

In this section, we provide additional results on real data analysis. Specifically, we calculate the Positive Predictive Values (PPV), the Negative Predictive Values (NPV), and the Saved Unnecessary Biopsies (SUB). The formula of the SUB is

$$\sum_s \left[rP\{d_s(\bar{\boldsymbol{Z}}_s) = 1, s < T \leqslant s + \tau \mid T > s\} - P\{d_s(\bar{\boldsymbol{Z}}_s) = 1, T > s + \tau \mid T > s\} \right],$$

where r is the specified cost-benefit ratio. The SUB is calculated as the difference between the count (or rate) of unnecessary biopsies patients can afford to catch an event, i.e., $rP\{d_0(\bar{\boldsymbol{Z}}_s) = 1, s < T \leqslant s + \tau \mid T > s\}$, and the count (or rate) of the unnecessary biopsies under the strategy $\boldsymbol{d}$, i.e., $P\{d_s(\bar{\boldsymbol{Z}}_s) = 1, T > s + \tau \mid T > s\}$. A positive SUB implies that the cost-benefit trade-off of the strategy $\boldsymbol{d}$ is better than the specified cost-benefit ratio r. Table 1 shows the summary of the results in terms of PPVs, NPVs, and SUBs. Overall, the proposed method has higher NPV but lower PPV. According to the SUBs, the cost-benefit trade-off is better for the proposed methods.

[Table 1 about here.]

Web Appendix E

In this section, we provide proof of Theorem 1. We first show the following lemma.

LEMMA 1: *For any k, s, and $k' < k$, we have*

$$\begin{aligned} & P\left\{d_s(\bar{\boldsymbol{Z}}_s) = a, s \leqslant T \leqslant s+\tau\right\} \\ = \; & P\left\{d_s(\bar{\boldsymbol{Z}}_s) = a, \Delta_{k'} = 0, \Delta_k = 1 \mid N_{k'} = s, N_k = s+\tau, \Omega(k,k')\right\}, \end{aligned}$$

where $\Omega(k,k')$ is an indicator of the event that the k-th biopsy and k'-th biopsy are adjacent observed biopsies, i.e., $\{\delta_k\zeta_k = 1, \delta_{k-1}\zeta_{k-1} = 0, \cdots, \delta_{k'+1}\zeta_{k'+1} = 1, \delta_{k'}\zeta_{k'} = 1\}$.

Proof. Notice that

$$\begin{aligned} & P\left\{d_s(\bar{\boldsymbol{Z}}_s) = a, \Delta_{k'} = 0, \Delta_k = 1 \mid N_{k'} = s, N_k = s+\tau, \Omega(k,k')\right\} \\ = \; & P\left\{d_s(\bar{\boldsymbol{Z}}_s) = a, T > N_{k'}, T \leqslant N_k \mid N_{k'} = s, N_k = s+\tau, \Omega(k,k')\right\} \\ = \; & P\left\{d_s(\bar{\boldsymbol{Z}}_s) = a, s < T \leqslant s+\tau \mid N_{k'} = s, N_k = s+\tau, \Omega(k,k')\right\}. \end{aligned}$$

Since $\boldsymbol{N}$ is a random vector as patients may visit at random times near the scheduled visits, i.e., the biopsy times $\boldsymbol{N}$ are independent of both T and $\{\boldsymbol{Z}_t\}_{t\in R_+}$; the $P(\delta_k = 1 \mid \boldsymbol{\Delta}, \boldsymbol{N}, \{\boldsymbol{Z}_t\}_{t\in R_+}) = \rho_k > 0$; the censoring indicator $P(\zeta_k = 1 \mid \boldsymbol{\Delta}, \boldsymbol{N}, \{\boldsymbol{Z}_t\}_{t\in R_+}) = \widetilde{\rho}_k > 0$, we have

$$P\left\{d_s(\bar{\boldsymbol{Z}}_s) = a, s < T \leqslant s+\tau \mid N_{k'} = s, N_k = s+\tau, \Omega(k,k')\right\} = P\left\{d_s(\bar{\boldsymbol{Z}}_s) = a, s < T \leqslant s+\tau\right\}.$$

Thus, we have

$$\begin{aligned} & P\left\{d_s(\bar{\boldsymbol{Z}}_s) = a, s \leqslant T \leqslant s+\tau\right\} \\ = \; & P\left\{d_s(\bar{\boldsymbol{Z}}_s) = a, \Delta_{k'} = 0, \Delta_k = 1 \mid N_{k'} = s, N_k = s+\tau, \Omega(k,k')\right\}. \end{aligned}$$

To show Theorem 1, we consider all the $\Omega(k,k')$'s with different k's. Notice that

$$
\begin{aligned}
& P\left\{d_s(\bar{\boldsymbol{Z}}_s)=a,\Delta_{(k)}=0,\Delta_k=1\mid N_{(k)}=s,N_k=s+\tau,\delta_k\zeta_k=1\right\}\\
= & \sum_{k'} P\left\{d_s(\bar{\boldsymbol{Z}}_s)=a,\Delta_{k'}=0,\Delta_k=1\mid N_{k'}=s,N_k=s+\tau,\Omega(k,k')\right\}\\
& \times P(\Omega(k,k')\mid N_{k'}=s,N_k=s+\tau)\\
= & P\left\{d_s(\bar{\boldsymbol{Z}}_s)=a,s\leqslant T\leqslant s+\tau\right\}\sum_{k'} P(\Omega(k,k')\mid N_{k'}=s,N_k=s+\tau)\\
= & P\left\{d_s(\bar{\boldsymbol{Z}}_s)=a,s\leqslant T\leqslant s+\tau\right\}.
\end{aligned}
$$

This concludes the proof of Theorem 1.

Web Appendix F

In this section, we provide proof of Theorem 2. To start with, we introduce some notations. Given a decision rule $\boldsymbol{d}$, we can define $\Phi(\boldsymbol{d};\xi)=J^{-1}\sum_j\left\{\mathrm{TPR}(d_{t_j};t_j)+\xi(t_j)\mathrm{FPR}(d_{t_j};t_j)\right\}$. To characterize theoretical property of the estimated decision rule $\widehat{d}_{\xi,\lambda_n}$, a generalization error compares $\Phi(\widehat{d}_{\xi,\lambda_n};\xi)$ with the optimal time-varying decision rule. The optimal time-varying decision rule is defined as the maximizers of $\mathrm{TPR}(d_{t_j};t_j)+\xi(t_j)\mathrm{FPR}(d_{t_j};t_j)$ for each j. Denote the maximizer for each j as $d^*_{\xi,j}$, and define $\Phi^*(\xi)=J^{-1}\sum_j\left\{\mathrm{TPR}(d^*_{\xi,j};t_j)+\xi(t_j)\mathrm{FPR}(d^*_{\xi,j};t_j)\right\}$. The generalization error is then defined as

$$
\Phi(\widehat{d}_{\xi,\lambda_n};\xi)-\Phi^*(\xi).
$$

To accommodate the case where ξ is chosen using the cost-benefit ratio, we derive an upper bound for the generalization error $\left\{\Phi(\widehat{d}_{\xi,\lambda_n};\xi)-\bar{\Phi}(\xi)\right\}$ which is uniformly held for $\xi\in\Xi=[\underline{\xi},\bar{\xi}]^J$. To estimate the decision rule, we employ the empirical risk minimization

$$
\min_{f\in\mathcal{F}}\ell_{\phi,n}(f;\xi,\lambda_n)=J^{-1}\sum_j E_n\left[W_{+,t_j}\phi\{f(\boldsymbol{Z}_{t_j})\}+W_{-,t_j}\phi\{-f(\boldsymbol{Z}_{t_j})\}\right]+\lambda_n\|f\|^2_{\mathcal{F}},
$$

where

$$
\begin{aligned}
W_{+,t_j} &= \left\{W_{1,t_j}-\xi(t_j)W_{-1,t_j+\tau}\right\}_+,\\
W_{-,t_j} &= \left\{W_{1,t_j}-\xi(t_j)W_{-1,t_j+\tau}\right\}_-,
\end{aligned}
$$

and $\mathcal{F}$ is a class of functions with a complexity constraint. In this work, the complexity constraint is related to the covering number of the space $\mathcal{F}$. The covering number $N\{\epsilon, \mathcal{F}, L_2(P)\}$ is defined as the minimal number of closed $L_2(P)$-balls of radius $\epsilon > 0$ required to cover $\mathcal{F}$, where $\|f\|^2_{P,2} = E(f^2)$ (Van de Geer, 2008). Denote the limit of $E\left(W_{+,t_j} \mid \boldsymbol{Z}_{t_j}\right)$ as $\overline{W}_{+,t_j}(\boldsymbol{Z}_{t_j})$, and $E\left(W_{-,t_j} \mid \boldsymbol{Z}_{t_j}\right)$ as $\overline{W}_{-,t_j}(\boldsymbol{Z}_{t_j})$ when $\max\{h, \widetilde{h}\} \to 0$. Under these notations, we assume the following conditions:

Assumption 1: Let ϕ be a convex function with $\phi'(0) < 0$. Denote

$$
\begin{aligned}
\eta_j(\boldsymbol{z}) \;&:=\; P(t_j < T \leqslant t_j + \tau \mid \boldsymbol{Z}_{t_j} = \boldsymbol{z})F^{-1}(t_j, t_j + \tau) \\
&\quad -\xi(t_j)P(T > t_j + \tau \mid \boldsymbol{Z}_{t_j} = \boldsymbol{z})F^{-1}(t_j + \tau, +\infty),
\end{aligned}
$$

where $F(s,t) = P(s < T \leqslant t)$. The surrogate loss ϕ satisfies that there exist constants $C > 0$ and $s \geqslant 1$ such that

$$
|\eta_j(\boldsymbol{z})|^s \leqslant C^s \left\{L_{\phi,j}(f;\xi) - L_{\phi,j}(f^*_{\xi,j};\xi)\right\},
$$

uniformly over all ξ's, $\boldsymbol{z}$'s, and j's, where

$$
\begin{aligned}
L_{\phi,j}(f;\xi) \;&=\; \phi\{f_j(\boldsymbol{Z}_{t_j})\}\overline{W}_{+,t_j}(\boldsymbol{Z}_{t_j}) + \phi\{-f_j(\boldsymbol{Z}_{t_j})\}\overline{W}_{-,t_j}(\boldsymbol{Z}_{t_j}), \\
f^*_{\xi,j} \;&=\; \arg\min_f L_{\phi,j}(f;\xi).
\end{aligned}
$$

Assumption 2: There exists constants $0 < v < 2$ and c such that $\forall \epsilon \in (0,1]$, we have $\sup_P \log N\{\epsilon, \mathcal{F}, L_2(P)\} \leqslant c\epsilon^{-v}$, where the supremum is taken over all finitely discrete probability measures P.

Assumption 3: The kernel function $K(\cdot)$ is a spherical symmetric univariate density function with a bounded 2nd order derivative and compact support; the kernel function $\widetilde{K}(\cdot,\cdot)$ is a spherical symmetric bivariate density function with a bounded 2nd order derivative and compact support.

Assumption 1 is related to the fisher consistency and excess risk. Bartlett et al. (2006)

shows that the condition $\phi'(0) < 0$ is a sufficient and necessary condition for the fisher consistency under a smooth choice of ϕ; in the Online Supporting Information (see Lemma 2), we show that the condition $|\eta_j(\boldsymbol{z})|^s \leqslant C^s \left\{L_{\phi,j}(f;\xi) - L_{\phi,j}(f^*_{\xi,j};\xi)\right\}$ can be used to derive an upper bound for the excess risk. Assumption 1 is satisfied for many common surrogate loss functions. For example, for $\phi(t) = (1-t)_+$, Assumption 1 is satisfied with $s = 1$; for a logistic loss or exponential loss, Assumption 1 is satisfied with $s = 2$.

To prove the main theorem, we first show a few useful lemmas. Lemma 2 shows the excess risk bound.

LEMMA 2: *Under Condition 1, the excess risk satisfies that*

$$\Delta\Phi(d;\xi) \leqslant J^{-1/s} C\{\Delta L_\phi(f;\xi)\}^{1/s},$$

where

$$\begin{aligned} \Delta\Phi(d;\xi) &= \Phi(d;\xi) - \Phi^*(\xi), \\ \Delta L_\phi(f;\xi) &= J^{-1}\sum_j E\left\{L_{\phi,j}(f;\xi)\right\} - L^*_\phi, \\ L^*_\phi &= J^{-1}\sum_{j=1}^{J} E\left\{L_{\phi,j}(f^*_{\xi,j};\xi)\right\}. \end{aligned}$$

Proof. First, we rewrite $\Phi(d;\xi)$ as

$$\begin{aligned} &\Phi(d;\xi) \\ =\ & J^{-1}\sum_j E\left[1\left\{d(\boldsymbol{Z}_{t_j}) = 1\right\} P\left(t_j < T \leqslant t_j + \tau \mid \boldsymbol{Z}_{t_j}\right)\right] F^{-1}(t_j, t_j+\tau) \\ & + J^{-1}\sum_j \xi(t_j) E\left[1\left\{d(\boldsymbol{Z}_{t_j}) = -1\right\} P\left(T > t_j + \tau \mid \boldsymbol{Z}_{t_j}\right)\right] F^{-1}(t_j+\tau, +\infty). \end{aligned}$$

Thus, we have that

$$\Delta\Phi(d;\xi) = J^{-1}\sum_j E\left[1\left\{d(\boldsymbol{Z}_{t_j}) \neq d^*_j(\boldsymbol{Z}_{t_j})\right\}\left|\eta_j(\boldsymbol{Z}_{t_j})\right|\right].$$

Next, we will show that

$$\sum_j 1\left\{d(\boldsymbol{Z}_{t_j}) \neq d^*_j(\boldsymbol{Z}_{t_j})\right\}\left|\eta_j(\boldsymbol{Z}_{t_j})\right| \leqslant \sum_j \left\{L_{\phi,j}(f;\xi) - L_{\phi,j}(f^*_{\xi,j};\xi)\right\}.$$

When $d(\boldsymbol{Z}_{t_j}) = d_j^*(\boldsymbol{Z}_{t_j})$, we have $1\left\{d(\boldsymbol{Z}_{t_j}) \neq d_j^*(\boldsymbol{Z}_{t_j})\right\} = 0$. Thus, naturally, the inequality holds. When $d(\boldsymbol{Z}_{t_j}) \neq d_j^*(\boldsymbol{Z}_{t_j})$, we have $1\left\{d(\boldsymbol{Z}_{t_j}) \neq d_j^*(\boldsymbol{Z}_{t_j})\right\}\left|\eta_j(\boldsymbol{Z}_{t_j})\right| \leqslant |\eta_j|$. Thus, by Hölder inequality, we have

$$\begin{aligned} & J^{-1}\sum_j |\eta_j| \\ \leqslant\ & (J^{-1}\sum_j |\eta_j|^s)^{1/s} \\ \leqslant\ & \{J^{-1}C^s\sum_j \Delta L_{\phi,j}(f;\xi)\}^{1/s} = J^{-1/s}C\left\{\sum_j \Delta L_{\phi,j}(f;\xi)\right\}^{1/s}. \end{aligned}$$

Taking the expectation on both sides, we have

$$\Delta\Phi(d;\xi) \leqslant J^{-1/s}C\{\Delta L_\phi(f;\xi)\}^{1/s},$$

uniformly holds for ξ's.

Lemma 3 shows that $\left|\phi\left(a\widehat{f}_{\xi,\lambda_n}\right)\right|$ can be uniformly bounded.

LEMMA 3: *For $a = 1$ or -1, we have*

$$\left|\phi\left(a\widehat{f}_{\xi,\lambda_n}\right)\right| \lesssim \lambda_n^{-1/2}, \quad \left|\phi\left(af^*_{\xi,\lambda_n}\right)\right| \lesssim \lambda_n^{-1/2},$$

uniformly hold for all $\xi \in [\underline{\xi},\bar{\xi}]^J$.

Proof. By the definition of $\widehat{f}_{\xi,\lambda_n}$, we have

$$\ell_{\phi,n}(f;\xi,\lambda_n) \leqslant \phi(0)J^{-1}\sum_j E_n\left\{\left|W_{1,t_j} - \xi(t_j)W_{-1,t_j+\tau}\right|\right\} \leqslant \phi(0)(1+\bar{\xi}).$$

Thus, we have

$$\|\widehat{f}_{\xi,\lambda_n}\|_{\mathcal{F}} \lesssim \lambda_n^{-1/2}.$$

Similarly, we can derive that $\|f^*_{\xi,\lambda_n}\|_{\mathcal{F}} \lesssim \lambda_n^{-1/2}$. By the Lipshcitz continuity of ϕ, we have

$$\left|\phi\left(a\widehat{f}_{\xi,\lambda_n}\right)\right| \lesssim \lambda_n^{-1/2}, \quad |\phi(af^*_{\xi,\lambda_n})| \lesssim \lambda_n^{-1/2}.$$

Lemma 4 provides an upper bound to the empirical mean of the difference in excess risk.

LEMMA 4: *Under Conditions 1- 3, we have*

$$\begin{aligned}\sup_{f\in\mathcal{B}(B_n),\xi\in[\underline{\xi},\bar{\xi}]} & \left|E_n\left\{J^{-1}\sum_j\left[\phi\left\{f(\boldsymbol{Z}_{t_j})\right\}\left(W_{+,t_j}-\overline{W}_{+,t_j}\right)\right.\right.\right. \\ & \left.\left.\left.+\phi\left\{-f(\boldsymbol{Z}_{t_j})\right\}\xi(t_j)\left(W_{-,t_j}-\overline{W}_{-,t_j}\right)\right]\right\}\right| \\ \lesssim\quad & C\lambda_n^{-1/2}\left\{h^2+(nh)^{-1/2}+\widetilde{h}^2+(n\widetilde{h}^2)^{-1/2}\right\},\end{aligned}$$

for a sufficient large C.

Proof. Notice that

$$\begin{aligned}& E_n\left\{J^{-1}\sum_j\left[\phi\left\{f(\boldsymbol{Z}_{t_j})\right\}\left(W_{+,t_j}-\overline{W}_{+,t_j}\right)+\phi\left\{-f(\boldsymbol{Z}_{t_j})\right\}\xi(t_j)\left(W_{-,t_j}-\overline{W}_{-,t_j}\right)\right]\right\} \\ = \;& (E_n-E)\left\{J^{-1}\sum_j\left[\phi\left\{f(\boldsymbol{Z}_{t_j})\right\}\left(W_{+,t_j}-\overline{W}_{+,t_j}\right)+\phi\left\{-f(\boldsymbol{Z}_{t_j})\right\}\xi(t_j)\left(W_{-,t_j}-\overline{W}_{-,t_j}\right)\right]\right\} \\ & +E\left\{J^{-1}\sum_j\left[\phi\left\{f(\boldsymbol{Z}_{t_j})\right\}\left(W_{+,t_j}-\overline{W}_{+,t_j}\right)+\phi\left\{-f(\boldsymbol{Z}_{t_j})\right\}\xi(t_j)\left(W_{-,t_j}-\overline{W}_{-,t_j}\right)\right]\right\}.\end{aligned}$$

Direct calculation yields

$$\begin{aligned}& \left|E\left\{J^{-1}\sum_j\left[\phi\left\{f(\boldsymbol{Z}_{t_j})\right\}\left(W_{+,t_j}-\overline{W}_{+,t_j}\right)+\phi\left\{-f(\boldsymbol{Z}_{t_j})\right\}\xi(t_j)\left(W_{-,t_j}-\overline{W}_{-,t_j}\right)\right]\right\}\right| \\ \lesssim\; & \lambda_n^{-1/2}\left(h^2+\widetilde{h}^2\right).\end{aligned}$$

Thus, we only need to bound the first term. To start with, we first consider

$$(E_n-E)\left\{J^{-1}\sum_j\left[\phi\left\{f(\boldsymbol{Z}_{t_j})\right\}W_{+,t_j}+\phi\left\{-f(\boldsymbol{Z}_{t_j})\right\}\xi(t_j)W_{-,t_j}\right]\right\}.$$

Let

$$\mathcal{F}_1=\left\{J^{-1}\sum_j\left[\phi\left\{f(\boldsymbol{Z}_{t_j})\right\}W_{+,t_j}+\phi\left\{-f(\boldsymbol{Z}_{t_j})\right\}\xi(t_j)W_{-,t_j}\right],f\in\mathcal{B}(B_n),\xi(t_j)\in[\underline{\xi},\bar{\xi}]\right\}.$$

The envelop function is given by

$$F_1=C\lambda_n^{-1/2}J^{-1}\sum_j\left(W_{+,t_j}+\bar{\xi}W_{-,t_j}\right).$$

By the uniform convergence rate of kernel estimation and direct calculation, $\|F_1\|_{P,2}=C\lambda_n^{-1/2}(h^{-1}+\bar{\xi}\widetilde{h}^{-2})$. By our entropy assumption, $\sup_P\log N(\epsilon,\mathcal{B}(B_n),L_2(P))\leqslant C(\epsilon/B_n)^{-v}$.

This implies that

$$\sup_p \log N(\epsilon \|F_1\|_{P,2}, \mathcal{F}_1, L_2(P)) \leqslant C\epsilon^{-\nu} - C\log\epsilon.$$

Thus,

$$J(1, \mathcal{F}_1, L_2) = \int_0^1 \left\{ \sup_p \log N(\epsilon\|F_1\|_{P,2}, \mathcal{F}_1, L_2(P)) \right\}^{1/2} d\epsilon \lesssim 1.$$

Thus, by the maximal inequality in Lemma 19.38 in Vaart (1998), we have that

$$\sup_{f \in \mathcal{B}(B_n), \xi \in [\underline{\xi}, \bar{\xi}]} |E_n - E| \left[J^{-1} \sum_j \phi \left\{ -f(\boldsymbol{Z}_{t_j}) \right\} \xi(t_j) \left(W_{-,t_j} - \overline{W}_{-,t_j} \right) \right] \lesssim n^{-1/2} \lambda_n^{-1/2} \bar{\xi} (h^{-1/2} + \widetilde{h}^{-1}).$$

We can show similar results to

$$|E_n - E| \left\{ J^{-1} \sum_j \left[\phi \left\{ f(\boldsymbol{Z}_{t_j}) \right\} \overline{W}_{+,t_j} + \phi \left\{ -f(\boldsymbol{Z}_{t_j}) \right\} \xi(t_j) \overline{W}_{-,t_j} \right] \right\}.$$

Now, we are ready to provide proof of the main theorem.

THEOREM 1: *Suppose that Conditions 1 - 3 hold with* $\lambda_n \to 0$*, with probability approaching to* 1*, we have that*

$$\Phi(\widehat{d}_{\xi,\lambda_n}; \xi) - \Phi^*(\xi) \lesssim J^{-1/s} C \left\{ \mathcal{A}(\lambda_n; \xi) + n^{-2/(v+2)} \lambda_n^{-v/(v+2)} + n^{-1}\lambda_n^{-1} + n^{-1}\lambda_n^{-1/2} + \lambda_n^{-1/2}\hbar \right\}^{1/s}$$

uniformly holds for all $\xi \in [\underline{\xi}, \bar{\xi}]^J$*, where*

$$\begin{aligned} \mathcal{A}(\lambda_n; \xi) &= \inf_{f \in \mathcal{F}} \left[\lambda_n \|f\|_{\mathcal{F}}^2 + J^{-1} \sum_j E\left\{ L_{\phi,j}(f; \xi) \right\} \right] - L_\phi^*, \\ \hbar &= h^2 + (nh)^{-1/2} + \widetilde{h}^2 + (n\widetilde{h}^2)^{-1/2}. \end{aligned}$$

Proof. [Proof of Theorem 1]

By Lemma 2, we have that

$$\Delta\Phi(\widehat{d}_{\xi,\lambda_n}; \xi) \leqslant J^{-1/s} C \{\Delta L_\phi(\widehat{f}_{\xi,\lambda_n}; \xi)\}^{1/s}.$$

Thus, it is sufficient to derive an upper bound for $\Delta L_\phi(\widehat{f}_{\xi,\lambda_n}; \xi)$.

By the definition of $\Delta L_\phi(f;\xi)$ and $\widehat{f}_{\xi,\lambda_n}$, we have

$$\begin{aligned}
&\Delta L_\phi(\widehat{f}_{\xi,\lambda_n};\xi)\\
=\;& J^{-1}\sum_j E\left\{L_{\phi,j}(\widehat{f}_{\xi,\lambda_n};\xi)\right\} - L_\phi^*\\
=\;& \left[J^{-1}\sum_j E\left\{L_{\phi,j}(\widehat{f}_{\xi,\lambda_n};\xi)\right\} + \lambda_n\|\widehat{f}_{\xi,\lambda_n}\|_{\mathcal{F}}^2 - J^{-1}\sum_j E\left\{L_{\phi,j}(f^*_{\xi,\lambda_n};\xi)\right\} - \lambda_n\|f^*_{\xi,\lambda_n}\|_{\mathcal{F}}^2\right]\\
& -L_\phi^* + J^{-1}\sum_j E\left\{L_{\phi,j}(f^*_{\xi,\lambda_n};\xi)\right\} + \lambda_n\|f^*_{\xi,\lambda_n}\|_{\mathcal{F}}^2\\
=\;& \lambda_n\|f^*_{\xi,\lambda_n}\|_{\mathcal{F}}^2 + \Delta L_\phi(f^*_{\xi,\lambda_n};\xi) + (I),
\end{aligned}$$

where

$$(I) = J^{-1}\sum_j E\left\{L_{\phi,j}(\widehat{f}_{\xi,\lambda_n};\xi)\right\} + \lambda_n\|\widehat{f}_{\xi,\lambda_n}\|_{\mathcal{F}}^2 - J^{-1}\sum_j E\left\{L_{\phi,j}(f^*_{\xi,\lambda_n};\xi)\right\} - \lambda_n\|f^*_{\xi,\lambda_n}\|_{\mathcal{F}}^2.$$

We now bound (I) using the empirical process theory. Let

$$\mathcal{L}_f = \left\{l_f = J^{-1}\sum_j L_{\phi,j}(\widehat{f}_{\xi,\lambda_n};\xi) + \lambda_n\|\widehat{f}_{\xi,\lambda_n}\|_{\mathcal{F}}^2 - J^{-1}\sum_j L_{\phi,j}(f^*_{\xi,\lambda_n};\xi) - \lambda_n\|f^*_{\xi,\lambda_n}\|_{\mathcal{F}}^2\right\},$$

where $f \in \mathcal{B}(B_n)$ is defined in Lemma 3. By Lemma 3, we have

$$\|l_f\|_\infty \lesssim \lambda_n^{-1/2}.$$

Following the proof of Theorem 3.4 in Zhao et al. (2012), we can show that there exists constant c such that $E(l_f^2) \leqslant cE(l_f)$ for all $l_f \in \mathcal{L}_f$, where

$$c = 2\lambda_n^{-1}\{C_L + 2(M\lambda_n)^{-1}\}^2.$$

Suppose that l_f satisfies $E_n(l_f) \leqslant \epsilon/2$ and $E(l_f) \geqslant \epsilon$ for some $\epsilon > 0$ to be chosen. We want to show that there exists some $l_{f'}$ such that $E_n(l_{f'}) \leqslant \epsilon/2$ and $E(l_{f'}) = \epsilon$. By the continuity of ϕ and the convexity of $\mathcal{B}(B_n)$ ($E(l_{f^*_{\xi,\lambda_n}}) = 0$), there is a $0 \leqslant t' \leqslant 1$ such that $f' = t'f + (1-t')f^*_{\xi,\lambda_n}$ and $E(l_{f'}) = \epsilon$. In addition, by the convexity of ϕ,

$$E_n(l_{f'}) \leqslant E_n(t'l_f) \leqslant t'\epsilon/2 \leqslant \epsilon/2.$$

To apply Lemma 6 in Bartlett et al. (2006), we need to verify that

$$E_n(l_{\widehat{f}_{\xi,\lambda_n}}) \leqslant \epsilon/2.$$

To show this, we have

$$\begin{aligned}
E_n(l_{\widehat{f}_{\xi,\lambda}}) &= E_n\left\{J^{-1}\sum_j L_{\phi,j}(\widehat{f}_{\xi,\lambda_n};\xi) + \lambda_n\|\widehat{f}_{\xi,\lambda_n}\|^2_{\mathcal{F}} - J^{-1}\sum_j L_{\phi,j}(f^*_{\xi,\lambda_n};\xi) - \lambda_n\|f^*_{\xi,\lambda_n}\|^2_{\mathcal{F}}\right\} \\
&= \ell_{\phi,j}(\widehat{f}_{\xi,\lambda_n};\xi,0) + \lambda_n\|\widehat{f}_{\xi,\lambda_n}\|^2_{\mathcal{F}} - \ell_{\phi,j}(f^*_{\xi,\lambda_n};\xi,0) - \lambda_n\|f^*_{\xi,\lambda_n}\|^2_{\mathcal{F}} \\
&\quad -E_n\left\{J^{-1}\sum_j\left[\phi\left\{\widehat{f}_{\xi,\lambda_n}(\boldsymbol{Z}_{t_j})\right\}\left(W_{+,t_j}-\overline{W}_{+,t_j}\right)\right.\right. \\
&\quad \left.\left.+\phi\left\{-\widehat{f}_{\xi,\lambda_n}(\boldsymbol{Z}_{t_j})\right\}\left(W_{-,t_j}-\overline{W}_{-,t_j}\right)\right]\right\} \\
&\quad -E_n\left\{J^{-1}\sum_j\left[\phi\left\{f^*_{\xi,\lambda_n}(\boldsymbol{Z}_{t_j})\right\}\left(W_{+,t_j}-\overline{W}_{+,t_j}\right)\right.\right. \\
&\quad \left.\left.+\phi\left\{-f^*_{\xi,\lambda_n}(\boldsymbol{Z}_{t_j})\right\}\left(W_{-,t_j}-\overline{W}_{-,t_j}\right)\right]\right\} \\
&\leqslant 0 + C\lambda_n^{-1/2}\left\{h^2 + (nh)^{-1/2} + \widetilde{h}^2 + (n\widetilde{h}^2)^{-1/2}\right\}.
\end{aligned}$$

where C is a sufficiently large constant. Thus, we have $E_n(l_{\widehat{f}_{\xi,\lambda_n}}) \leqslant \epsilon/2$ with $\epsilon \geqslant C\lambda_n^{-1/2}\hbar$, where $\hbar = h^2+(nh)^{-1/2}+\widetilde{h}^2+(n\widetilde{h}^2)^{-1/2}$. Therefore, Lemma 6 in Bartlett et al. (2006) implies that with probability tending to one,

$$E(l_{\widehat{f}_{\xi,\lambda_n}}) \leqslant \epsilon,$$

provided that

$$\epsilon \geqslant \max\left\{\epsilon^*, C\lambda_n^{-1}n^{-1}, C\lambda_n^{-1/2}n^{-1}, C\lambda_n^{-1/2}\hbar\right\},$$

where C is a sufficient alrge constant and $\epsilon^* \geqslant 12\xi_{\mathcal{L}_f}(\epsilon^*)$. It remains to find ϵ^*.

Define $\mathcal{G}_f = \{E(l_f) - l_f : E(l_f) = \epsilon, l_f \in \mathcal{L}_f\}$. Let $Z = \sup_{g_f\in\mathcal{G}_f} E_n(g_f)$, then by definition of $\xi_{\mathcal{L}_f}$ (see Lemma 6 in Bartlett et al. (2006)),

$$\xi_{\mathcal{F}_f}(\epsilon) = E(Z) = E\left\{\sup_{g_f\in\mathcal{G}_f} E_n(g_f)\right\} = E\left\{\sup_{E(l_f^2)\leqslant c\epsilon} |E(l_f) - E_n(l_f)|\right\}.$$

Because $f \in \mathcal{B}(B_n)$, there exists a constant depending on v, so that $\sup_P \log N(\epsilon, \mathcal{B}(B_n), L_2(P)) \leqslant$

$C_v(\epsilon/B_n)^{-v}$. It follows that

$$\begin{aligned}\log N(\epsilon, \mathcal{L}_f, L_2(P)) &\leqslant \log N(\epsilon, \mathcal{B}(B_n), L_2(P)) \\ &\quad + \log N(\epsilon, \{\lambda_n \|f\|^2, f \in \mathcal{B}(B_n)\}, L_2(P)) \\ &\quad + \log N(\epsilon, \{\phi(-f)\xi \overline{W}_{-,t_j}, f \in \mathcal{B}(B_n), \xi \in \{\xi \in [\underline{\xi}, \bar{\xi}]\}\}, L_2(P)) \\ &\leqslant CB_n^v \epsilon^{-v} + \log(M^2/\epsilon) + \log(B_n/\epsilon) \\ &\leqslant 3CB_n^v \epsilon^{-v}.\end{aligned}$$

Hence, $E(Z)$ is bounded above by

$$3C \max\left\{n^{-2/(v+2)}\lambda_n^{-v/(v+2)}, n^{-1/2}\lambda_n^{-v/4}\epsilon^{(2-v)/4}\right\},$$

by Proposition 5.5 in Steinwart and Scovel (2007). Consequently, it suffices to choose

$$\epsilon^* = 3Cn^{-2/(v+2)}\lambda_n^{-v/(v+2)},$$

and by solving $\epsilon \geqslant 12\xi_{\mathcal{L}_f}(\epsilon)$ for ϵ^*. Therefore, we have

$$\epsilon \geqslant \max\left\{3Cn^{-2/(v+2)}\lambda_n^{-v/(v+2)}, C\lambda_n^{-1}n^{-1}, C\lambda_n^{-1/2}n^{-1}, C\lambda_n^{-1/2}\hbar\right\}.$$

This competes the proof.

Corollary 1: *Under the assumption that* $|\Phi^*(\xi_1) - \Phi^*(\xi_2)| \leqslant C\|\xi_1 - \xi_2\|_\infty^\alpha$, *with probability approaching to* 1, *we have that*

$$\begin{aligned}&\Phi(\widehat{d}_{\widehat{\xi},\lambda_n}; \widehat{\xi}) - \Phi^*(\xi^*) \\ \lesssim\ & C\hbar^\alpha + J^{-1/s}C\left\{\sup_\xi \mathcal{A}(\lambda_n; \xi) + n^{-2/(v+2)}\lambda_n^{-v/(v+2)} + \lambda_n^{-1}n^{-1} + \lambda_n^{-1/2}n^{-1} + \lambda_n^{-1/2}\hbar\right\}^{1/s}.\end{aligned}$$

Proof. The proof is immediate from Theorem 1.

References

Bartlett, P. L., Jordan, M. I., and Mcauliffe, J. D. (2006). Convexity, classification, and risk bounds. *Journal of the American Statistical Association* **101,** 138–156.

Chan, S., Wang, X., Jazić, I., Peskoe, S., Zheng, Y., and Cai, T. (2021). Developing and evaluating risk prediction models with panel current status data. *Biometrics* **77,** 599–609.

Dong, X., Zheng, Y., Lin, D. W., Newcomb, L., and Zhao, Y.-Q. (2023). Constructing time-invariant dynamic surveillance rules for optimal monitoring schedules. *Biometrics* **79,** 3895–3906.

Silverman, B. W. (2018). *Density estimation for statistics and data analysis.* Routledge.

Steinwart, I. and Scovel, C. (2007). Fast rates for support vector machines using gaussian kernels. *The Annals of Statistics* **35,** 575–607.

Vaart, A. W. v. d. (1998). *Asymptotic Statistics.* Cambridge Series in Statistical and Probabilistic Mathematics. Cambridge University Press.

Van de Geer, S. A. (2008). High-dimensional generalized linear models and the lasso. *The Annals of Statistics* **36,** 614–645.

Zhao, Y., Zeng, D., Rush, A. J., and Kosorok, M. R. (2012). Estimating individualized treatment rules using outcome weighted learning. *Journal of the American Statistical Association* **107,** 1106–1118. PMID: 23630406.

Received October 2007. *Revised February* 2008. *Accepted March* 2008.

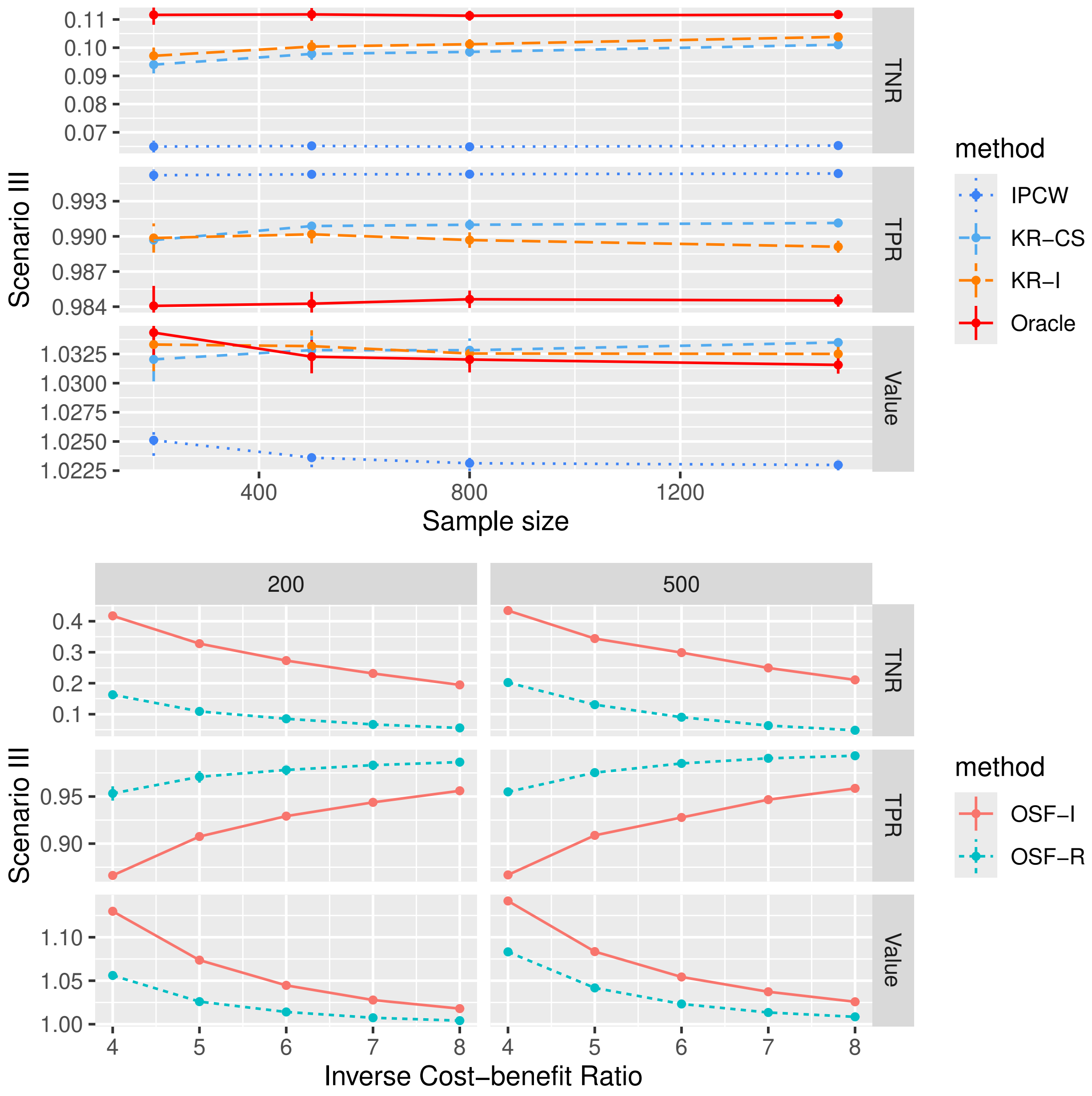

Figure 1. Upper: Estimating TNR, TPR, and weighted benefits value of a derived tailored AS rule using IPCW method in Dong et al. (2023) ('IPCW'), the method proposed in Chan et al. (2021) ('KS-CS'), and our proposed method ('KS-I'). The lines labeled "Oracle" are the TPRs, TNRs, and values calculated using the true event time without censoring. Lower: TNR, TPR, and weighted benefits value achieved by different methods. The x-axis represents the inverse cost-benefit ratio, r, i.e., the acceptable number of unnecessary biopsies to perform to catch an event (disease progression).

Table 1
Comparisons using the PASS and UCSF data on additional metrics.

PASS Only						
r		4	6	8	10	12
OSF-I	PPV	0.149 (0.140,0.158)	0.128 (0.122,0.134)	0.122 (0.117,0.128)	0.119 (0.114,0.125)	0.118 (0.112,0.123)
	NPV	0.951 (0.947,0.956)	0.961 (0.953,0.969)	0.939 (0.910,0.969)	0.917 (0.881,0.954)	0.905 (0.866,0.944)
	SUB	0.041 (0.032,0.050)	0.261 (0.252,0.270)	0.548 (0.540, 0.555)	0.856 (0.850, 0.862)	1.176 (1.169, 1.183)
OSF-R	PPV	0.178 (0.150,0.206)	0.234 (0.212,0.237)	0.235 (0.222,0.249)	0.224 (0.210,0.238)	0.199 (0.188,0.211)
	NPV	0.888 (0.883,0.894)	0.898 (0.893,0.903)	0.906 (0.901,0.911)	0.916 (0.912,0.921)	0.927 (0.923,0.930)
	SUB	0.017 (0.012,0.022)	0.116 (0.102,0.131)	0.316 (0.295,0.336)	0.596 (0.571,0.621)	0.904 (0.874,0.933)
PASS Train + UCSF Test						
r		4	6	8	10	12
OSF-I	PPV	0.171 (0.151,0.333)	0.167 (0.146,0.319)	0.163 (0.145,0.320)	0.163 (0.144,0.320)	0.163 (0.144,0.320)
	NPV	0.887 (0.796,0.926)	0.881 (0.764,0.920)	0.889 (0.747,0.928)	0.903 (0.804,0.976)	0.899 (0.782,0.978)
	SUB	0.193 (0.094,0.585)	0.593 (0.416,1.126)	1.081 (0.813,1.799)	1.646 (1.243,2.500)	1.996 (1.645,3.129)
OSF-R	PPV	0.023 (0.118,0.664)	0.234 (0.158,0.440)	0.197 (0.167,0.381)	0.186 (0.178,0.396)	0.181 (0.163,0.378)
	NPV	0.842 (0.738,0.863)	0.856 (0.759,0.875)	0.857 (0.761,0.880)	0.863 (0.772,0.892)	0.870 (0.787,0.897)
	SUB	-0.002 (-0.006,0.095)	0.234 (0.158,0.516)	0.540 (0.428,1.139)	0.921 (0.707,1.574)	1.425 (1.143,2.486)
